# Anti-Rheumatic Properties of Gentiopicroside Are Associated With Suppression of ROS-NF-κB-NLRP3 Axis in Fibroblast-Like Synoviocytes and NF-κB Pathway in Adjuvant-Induced Arthritis

**DOI:** 10.3389/fphar.2020.00515

**Published:** 2020-05-04

**Authors:** Meiling Wang, Hongyan Li, Yanfang Wang, Yanfei Hao, Yanan Huang, Xinlin Wang, Yongying Lu, Yuan Du, Fenghua Fu, Wenyu Xin, Leiming Zhang

**Affiliations:** ^1^School of Pharmacy, Key Laboratory of Molecular Pharmacology and Drug Evaluation, Ministry of Education, Yantai University, Yantai, China; ^2^Department of Orthopedics and Traumatology, Yantaishan Hospital, Yantai, China; ^3^School of Pharmacy, Key Laboratory of Prescription Effect and Clinical Evaluation of State Administration of Traditional Chinese Medicine of China, Binzhou Medical University, Yantai, China

**Keywords:** gentiopicroside, adjuvant-induced arthritis, rheumatoid arthritis fibroblast-like synoviocytes, reactive oxygen species, nuclear factor-kappa B, NLRP3 inflammasome

## Abstract

Rheumatoid arthritis (RA) is among the most prevalent forms of autoimmunity. Gentiopicroside (Gent) is an iridoid glucoside derived from the *Gentiana Macrophylla Pall* which is used in traditional Chinese medicine to treat RA. The present study was designed to explore the ability of Gent to combat RA and to explore the molecular basis for such anti-RA activity both *in vitro* using tumor necrosis factor alpha (TNF-α)-stimulated human RA fibroblast-like synoviocytes (RA-FLS) and *in vivo* using a rat adjuvant-induced arthritis (AIA) model. We found that Gent was able to significantly reduce the swelling of joints and arthritic index scores, with corresponding reductions in synovial inflammatory cell infiltration, synovial hyperplasia, and bone erosion in treated AIA rats. Importantly, Gent 200 mg/kg reduced thymus index in AIA rats, but had no effect on spleen index and body weight, it revealed that Gent was relatively safe at the dose we chose. We further found that Gent was able to suppress the TNF-α-induced proliferation and migration of RA-FLS cells. This suppression was attributed to the ability of Gent to block NOD-like receptor protein 3 (NLRP3), apoptosis-associated speck-like protein containing a CARD (ASC), and caspase-1, thereby disrupting the activation of the NLRP3 inflammasome. Consistent with such suppression, Gent led to a significant decrease in IL-1β secretion by treated cells. Furthermore, this reduction in NLRP3 inflammasome activation was also associated with decreases in the activation of nuclear factor (NF-κB), the production of reactive oxygen species (ROS), and the expression of inflammatory IL-6. Together these findings indicate that Gent can suppress the ROS-NF-κB-NLRP3 axis to alleviate RA symptoms.

**Chemical compounds studied in this article:**

Gentiopicroside (PubChem CID: 88708).

## Graphical Abstract

Overview of the mechanistic basis for Gent-mediated treatment of rheumatoid arthritis.

**Graphical Abstract:**
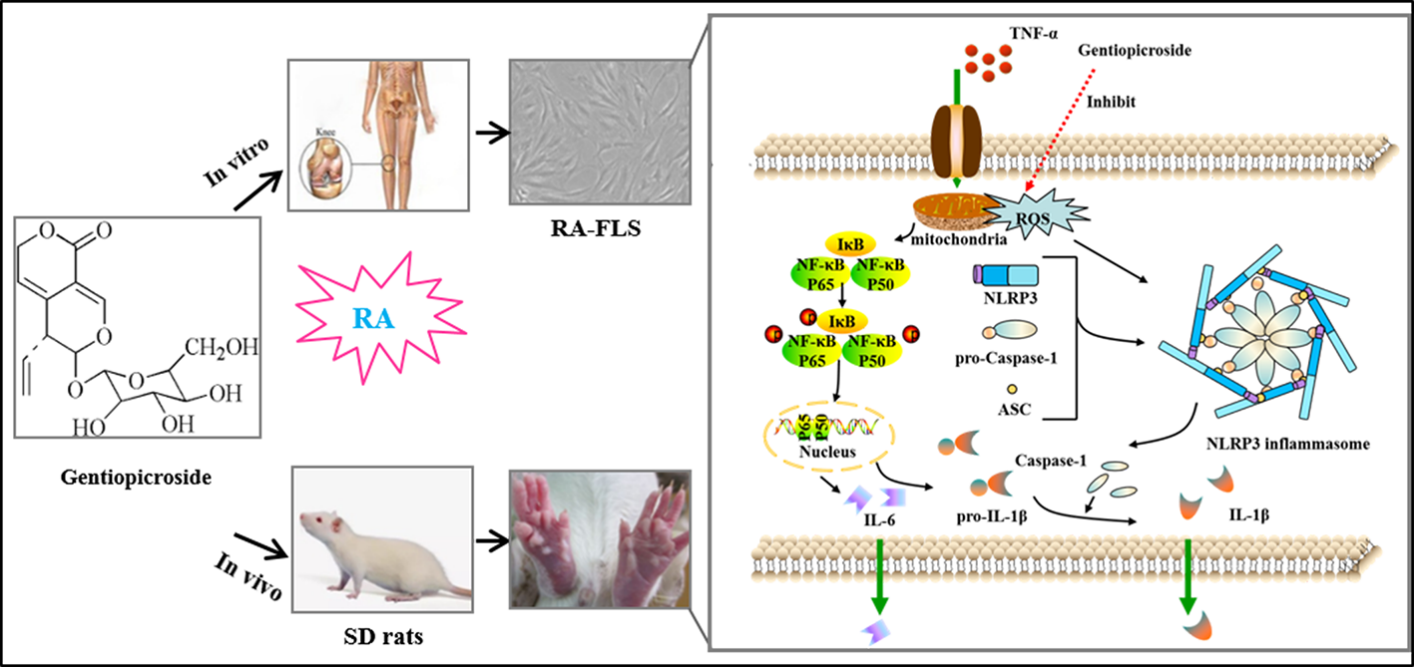
Overview of the mechanistic basis for Gent-mediated treatment of rheumatoid arthritis. Stimulation of cells with TNF-α leads to both NF-κB activation and ROS production, thereby driving NLRP3, ASC, pro-caspase-1, and pro-IL-1β upregulation. In response to the above signal, the NLRP3 inflammasome then undergoes assembly and cleavage of pro-caspase-1 to yield an active form of this protein, thus forming the NLRP3 inflammasome which then cleaves pro-IL-1β into mature IL-1β that is subsequently secreted from stimulated cells. This IL-1β subsequently promotes RA progression. Gent was able to inhibit ROS-NF-κB activation and to disrupt NLRP3 inflammasome assembly, thereby reducing IL-1β secretion. This in turn resulted in decreased RA-FLS migration and proliferation, thus significantly decreasing the severity of RA immunopathological injury.

## Introduction

Rheumatoid arthritis (RA) is a chronic and highly debilitating form of systemic autoimmunity associated with the progressive destruction of bone and cartilage, systemic synovitis, synovial lining thickening, and inflammatory cell infiltration of the subintimal layer (Schett and Gravallese, 2012). RA incidence is increasing globally, affecting between 0.5% and 1% of the overall population with a 3:1 female-to-male ratio (Safiri et al., 2019).

The progression and etiology of RA can vary widely between affected individuals, and its treatment remains challenging. Significant improvements in the quality of life of RA patients have been achieved *via* the administration of compounds including non-steroidal anti-inflammatory drugs (NSAIDs), glucocorticoids (GCs) and biologics including anti-tumor necrosis factor alpha (TNF-α) and immune costimulatory proteins (Scott et al., 1998). Despite substantial improvements in the treatment of RA in the past 10 years, up to 30% of patients either do not respond to these interventions or suffer significant treatment-related side effects (Rutherford et al., 2018).

The formation of an inflamed and hyperplasic synovial lining referred to as a pannus is considered to be a pathological hallmark of RA. This pannus is composed of macrophages, neutrophils, and other inflammatory cell types that can secrete TNF-α. In addition, fibroblast-like synoviocytes (FLS) within the pannus are responsive to paracrine TNF-α production, resulting in an inflammatory cell-TNF-α-FLS axis that can drive disease progression (Firestein, 2003; Noss and Brenner, 2008). When activated, FLS exhibit tumor-like proliferation and aggressive migration, resulting in synovial hyperplasia and consequent bone destruction (Shang et al., 2016). FLS can also indirectly drive bone degradation *via* secreting inflammatory factors including IL-1β, IL-8, and IL-6 (Garcia-Carbonell et al., 2016). Therapeutically targeting and inhibiting these aggressive proinflammatory FLS activities, thus, has the potential to alleviate RA symptoms and to arrest disease progression. Nuclear factor-kappa B (NF-κB) is well established as a key inflammatory regulator of RA. This transcription factor is highly activated in RA, resulting in substantial pathogenic production of IL-6 and TNF-α (Zhou et al., 2014). In addition to NF-κB, the NOD-like receptor protein 3 (NLRP3) inflammasome, which is a multimeric protein complex of NLRP3, apoptosis-associated speck-like protein containing (ASC), and caspase-1 (Wang et al., 2018), is thought to function as a key regulator of RA. For example, work by Zhao et al. suggests that NLRP3 inflammasome activity controls Th17 cell differentiation in humans suffering from RA (Zhao et al., 2018). Mice lacking expression of either NLRP3 or caspase-1 have further been shown to be protected from the induction of experimental arthritis (Vande-Walle et al., 2014).

**Figure 1:**
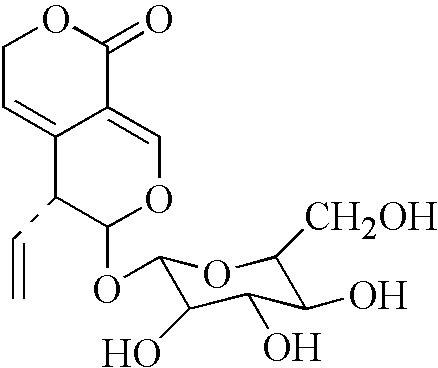
Chemical structure of Gent.

*Gentiana Macrophylla Pall* is a plant that is used for the treatment of RA in traditional Chinese medicine. The active ingredients within this plant and the mechanisms underlying their anti-RA activity, however, remain to be determined. Gentiopicroside (Gent) (**Figure 1**) is an iridoid glucoside and one of the primary compounds enriched in *Gentiana Macrophylla Pall* roots (Jia et al., 2016). Gent has been shown to possess analgesic (Liu et al., 2016), anti-inflammatory (Zhao et al., 2015; Chen et al., 2018), anti-cancer (Li et al., 2019), lipid regulating (Choi et al., 2019), and antidepressant properties (Deng et al., 2018). Gent has been shown to mediate protective efficacy on joints in the adjuvant-induced arthritis (AIA) model (Xie et al., 2019) and reduce the secretion of inflammatory factors in FLS (Zhang et al., 2019). However, the effects of Gent on the abnormal proliferation and migration of FLS have not been reported, and the mechanism *in vivo* and *in vitro* has not been studied in depth.

This study, therefore, sought to assess whether Gent was able to inhibit experimental arthritis *in vivo* and affect functions of RA-FLS *in vitro*, and to clarify the molecular basis for the anti-RA activity of Gent.

## Materials and Methods

### Reagents and Drugs

Gentiopicroside (Molecular formula: C_16_H_20_O_9_, Molecular weight: 356.32 g·mol^−1^, batch no: GR-135-180320, purity ≥ 98%) was from Nanjing Guangrun Biotechnology Co., Ltd. (Nanjing, China). Dexamethasone tablets (Dex, batch no: 160601201) were from Chen Xin Pharmaceutical Co., Ltd. (Jining, China). Dexamethasone powder (D4902, purity ≥ 97%) was from Sigma-Aldrich (MO, USA). Complete Freund’s adjuvant (CFA, 7027) was from Chondrex, Inc. (MA, USA). Recombinant human TNF-α (P00029), and IL-6 (SEKH-0013) and IL-1β (SEKH-0002) ELISA kits were from Beijing Solarbio Science & Technology Co., Ltd. (Beijing, China). The Cell Light™ EdU Apollo^®^488 In Vitro Imaging Kit (C10310-3) was from RiboBio (Guangzhou, China). The Reactive Oxygen Species Kit (S0033) was from Beyotime Institute of Biotechnology (Haimen, China). Antibodies specific for IκBα (L35A5), p-IκBα (2859S), NF-κB p65 (8242S), p-p65 (3033S), Vimentin (5741T), NLRP3 (15101S), and ASC (67824S) were from Cell Signaling Technology (MA, USA). Anti-CD68 (NB100-683SS) was from Novus Biologicals (CO, USA). Anti-caspase-1 (NBP1-76605) was from RD system (MN, USA). All other chemicals were analytical grade.

### Animals

Male Sprague-Dawley rats (160–180 g) were obtained from Jinan Pengyue Experimental Animal Breeding Co., Ltd. [Qualiﬁed No. SCXK (Lu) 20140007, Jinan, China] and were housed in a 24 ± 1°C facility with 55 ± 5% relative humidity and a 12-h light/dark cycle. Animals had free food and water access and were allowed to acclimate for 3 days prior to experimentation. The Animal Ethics Committee of the Yantai University approved all animal studies described herein, which were consistent with the National Institutes of Health Guide for the Care and Use of Laboratory Animals.

### AIA Induction

**Figure 2:**
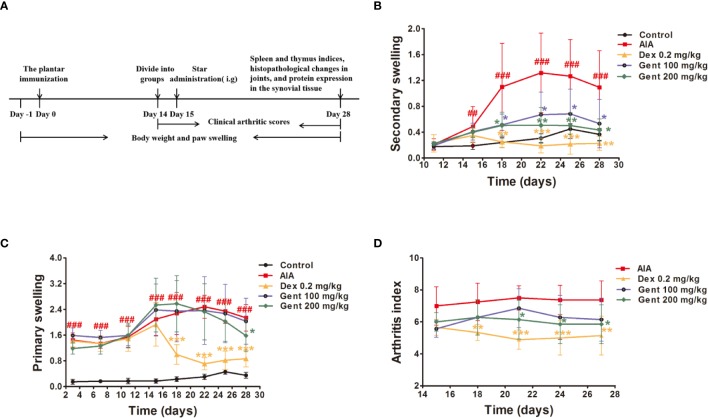
Gent disrupts AIA progression. **(A)** AIA model development and treatment timeline. **(B**, **C)** Primary and secondary paw swelling were measured *via* plethysmometer. **(D)** Clinical arthritic scores were used to assess disease severity. Data were presented as mean ± SD (*n* = 10 for control group, 8 for AIA group, 9 for Dex 0.2 mg/kg group, 7 for Gent 100 mg/kg group, 7 for Gent 200 mg/kg group). ^##^*P* < 0.01, ^###^*P* < 0.001 vs. control; *P < 0.05, **P < 0.01, and ***P < 0.001 vs. AIA group.

The AIA treatment regimen in the present study is shown in **Figure 2A**. Briefly, rats received an intradermal injection with 0.1 mL of either CFA or PBS (as a control) at the voix pedis of the right hind limb as in previous studies (Cai et al., 2006). The day of this initial immunization was considered to be day 0 for experimental purposes.

### Drug Administration

Rats were randomly separated into 5 different experimental groups: control, AIA, Dex 0.2 mg/kg, Gent 100 mg/kg, and Gent 200 mg/kg groups. Intragastric Dex and Gent administration was conducted once per day on days 15 to 28 post-immunization. Control and AIA group animals were instead intragastrically administered 0.5% sodium carboxymethyl cellulose (CMC-Na) on the same schedule. Gent concentrations used in this study were based on both prior studies and pilot experiments.

### Paw Swelling and Body Weight Measurements

A plethysmometer (YLS-7A, Shandong Academy of Medical Sciences, China) was used to measure paw swelling while an electronic scale was used for body weight measurements. Briefly, the right paw of each animal was measured every 3 to 4 days, with measurements of the left paw beginning on day 14 as a measure of secondary swelling. Each paw was measured 3 times, and the average measurement was recorded. Rat body weight was measured every 3 days.

### Arthritic Index Scoring

Two researchers blinded to study design independently evaluated and scored arthritic severity in study animals. A 0 to 4-point-based scale was used to assess arthritic index in these animals as follows: 0, normal; 1, slight erythema and swelling; 2, moderate erythema and swelling; 3, severe erythema and swelling with limited joint usage; and 4, deformed or ankylosed paws (Lee et al., 2018). Both hind limbs were scored in each animal for a maximum possible score of 8.

### Immune Organ Index

On day 28 post-immunization, 10% chloral hydrate was used to euthanize all animals. The spleen and thymus of each animal were then excised and weighed. Immune organ index values were then established as the ratio of wet thymus or spleen weight to overall body weight (mg/g).

### Histological Analysis

Following chloral hydrate anesthetization and cervical dislocation on day 28 post-immunization, the ankle joints of study animals were collected, fixed using 10% neutral formalin, decalcified over a 4-week period using 20% EDTA, dehydrated, and paraffin embedded. Longitudinal 5-μm-thick sections of these joints were then prepared in the sagittal plane and subjected to hematoxylin & eosin (HE) staining in order to assess general tissue histology. Histologic scores were then assigned to samples based upon inflammatory cell infiltration, pannus formation, synovial hyperplasia, and bone/cartilage destruction, as in previous reports (Quan et al., 2014).

### Primary RA-FLS Culture

Discarded synovial tissue samples isolated from 1 human RA patients undergoing synovectomy were provided as a gift by the Yantaishan Hospital in Yantai, China. All patients had a confirmed RA diagnosis as defined according to the American College of Rheumatology 1987 revised criteria. These human experiments were conducted in accordance with the Declaration of Helsinki.

Following collection, synovial tissue was minced into 1 mm^3^ pieces prior to digestion for 2 h using 0.4% type I collagenase and for 30 min using 0.25% trypsin, with both digestions being carried out in a 37°C 5% CO_2_ incubator. After digestion, a 70-μm strainer was used to filter cells, which were then washed thrice in PBS. The resultant FLS were then cultured in DMEM/F12 containing 10% FBS at 37°C in a 5% CO_2_ incubator. Light microscopy was used to confirm FLS morphology, while anti-vimentin and anti-CD68 immunofluorescent staining were used to confirm cell purity. Cells that had been passaged 4 to 8 times were used for all studies (Toh et al., 2005).

### Cell Viability Assay

An MTT assay was used to assess FLS viability. Briefly, 5 × 10^3^ FLS cells were plated per well of a 96-well plate. Following a 2-h starvation period, cells were treated with a range of Gent (25–200 μM) and Dex (25–200 nM) concentrations for a 72-h period. Next, 20-μL MTT (5 mg/mL) was added per well, and cells were incubated for 4 h. Supernatants were then discarded and 150 μL DMSO was added per well. After a 10-min incubation with shaking to dissolve crystals, absorbance values at 570 nm in each well were measured *via* microplate reader (Thermo Fisher Scientific).

### Cell Proliferation Assays

In addition to their use for measuring cell viability, MTT assays were also used to establish the ability of Gent to modulate TNF-α-induced RA-FLS cell proliferation. For these experiments, cells were pre-treated with a range of Gent (25–200 μM) and Dex (25–200 nM) concentrations for 2 h prior to the addition of TNF-α (10 ng·mL^−1^) for 72 h.

As another measure of FLS proliferation, incorporation of the thymidine analog 5-Ethynyl-2′-deoxyuridine (EdU) into proliferating cells was quantified. Briefly, cells were incubated with EdU (10 μM) for 48 h prior to a 24-h stimulation with TNF-α. Paraformaldehyde was then used to fix cells, which were then subjected to permeabilization using 0.5% Triton X-100 and staining with an Apollo^®^ staining reaction solution. Cellular nuclei were then stained using Hoechst 33342. A Cellomics ArrayScan VTI HCS Reader (Thermo Fisher Scientific) was used to image cells, with the number of EdU-positive cells being quantified.

### Wound Healing Assay

To assess wound healing, 4.5 × 10^4^ RA-FLS were added per well of a 6-well plate and were grown until confluent, at which time a sterile 200 μL micropipette tip was used to generate a scratch wound in the monolayer surface. PBS was used to remove cellular debris, and fresh DMEM/F12 media was then added. Appropriate cell wells had been pretreated with Dex or Gent for 2 h prior to TNF-α stimulation. Scratch wound area was imaged inverted phase-contrast microscope (Olympus Corp., Tokyo, Japan) after 0 and 48 h (Nakamura et al., 2018).

### Cytokine Measurements

In order to assess cytokine production, 5 × 10^3^ RA-FLS were plated per well of 96-well plates, and were pretreated for 2 h using Dex or Gent prior to stimulation with TNF-α for 48 h. Supernatant IL-6 and IL-1β concentrations were measured using ELISA kits based on provided directions.

### Intracellular ROS Detection

DCFH-DA was used to measure intracellular ROS levels in RA-FLS cells in the present study. Briefly, 5 × 10^3^ RA-FLS were plated per well of 96-well plates, and were pretreated for 2 h using Dex or Gent prior to stimulation with TNF-α for 48 h. Cells were then incubated for 30 min in DMEM/F12 supplemented with 10 µM DCFH-DA, after which DAPI was used to stain cell nuclei for 30 min. A Cellomics ArrayScan V^TI^ HCS Reader was then used to measure ROS levels within cells.

### Immunofluorescence Microscopy

Cell purity, NLRP3 and caspase-1 expression, and p65 nuclear translocation were all analyzed *via* immunofluorescence microscopy. Briefly, 5 × 10^3^ RA-FLS were plated per well of 96-well plates, and were pretreated for 2 h using Dex or Gent prior to stimulation with TNF-α for either 20 min or 48 h. Cells were then fixed for 30 min using 4% paraformaldehyde, after which they were permeabilized for 15 min with 0.3% Triton X-100, blocked for 30 min using 1% BSA, and probed at 4°C overnight using antibodies specific for vimentin, CD68, p65, NLRP3, and caspase-1 (1: 200). Cells were then probed with fluorescent secondary antibodies, stained using DAPI, and visualized with a Cellomics ArrayScan VTI HCS Reader (Karonitsch et al., 2018).

### Western Blotting

4.5 × 10^4^ RA-FLS were plated per well of 6-well plates, and were pretreated for 2 h using Dex or Gent prior to stimulation with TNF-α for 48 h.

A lysis buffer supplemented with protease inhibitors was then used to extract total protein from these cells or rat synovial tissue samples. Protein levels in these lysates were quantified with a BCA Protein Assay Kit. A total of 30 μg of protein per sample was then separated *via* 10% SDS-PAGE prior to transfer onto a PVDF membrane that was blocked for 2 h using 5% non-fat milk and probed overnight with antibodies specific for IκBα, p-IκBα, p65, p-p65, NLRP3, ASC, and caspase-1 (1: 1000) at 4°C. Blots were then probed for 1 h with appropriate HRP-conjugated secondary antibodies, followed by detection of protein bands *via* enhanced chemiluminescence (ChemiDoc™ XRS, Bio-Rad, Shanghai, China) (Rebsamen et al., 2015).

### Statistical Analysis

Data were presented as mean ± standard deviation (SD). The Quantity One 1-D 4.6.2 analysis software (Bio-Rad) was used for semi-quantitative protein measurements. ImageJ v1.47 (NIH, MD, USA) was used to quantify cellular migration. The Colunbus software was used for quantification of mean fluorescence intensity. Differences among experimental groups were compared *via* ANOVAs with Tukey’s *post hoc* test using GraphPad Prism 6.0 (CA, USA). *P* < 0.05 was the significance threshold.

## Results

### Gent Impacts Paw Swelling and Arthritic Index Values in AIA Rats

Beginning 15 days post-immunization, AIA model rats exhibited multi-joint inflammation characteristic of arthritis that peaked after 22 days. Animals treated with both Dex and Gent reduced secondary swelling as compared to those animals in the AIA model group (**Figure 2B**). Dex significantly reduced primary swelling, similarly, Gent 200 mg/kg was also associated with significant reductions in primary swelling in treated animals only on day 28 (**Figure 2C**). AIA model animals exhibited arthritic symptoms such as swelling, erythema, nodule formation, ankyloses, and hind paw deformities starting on day 15 post-immunization. Treatment with Dex and Gent 200 mg/kg were able to not only reduce paw swelling but also to decrease arthritic index values in rats with established disease (**Figure 2D**).

### Gent Alters AIA-Associated Histopathological Changes in Rat Joints.

**Figure 3:**
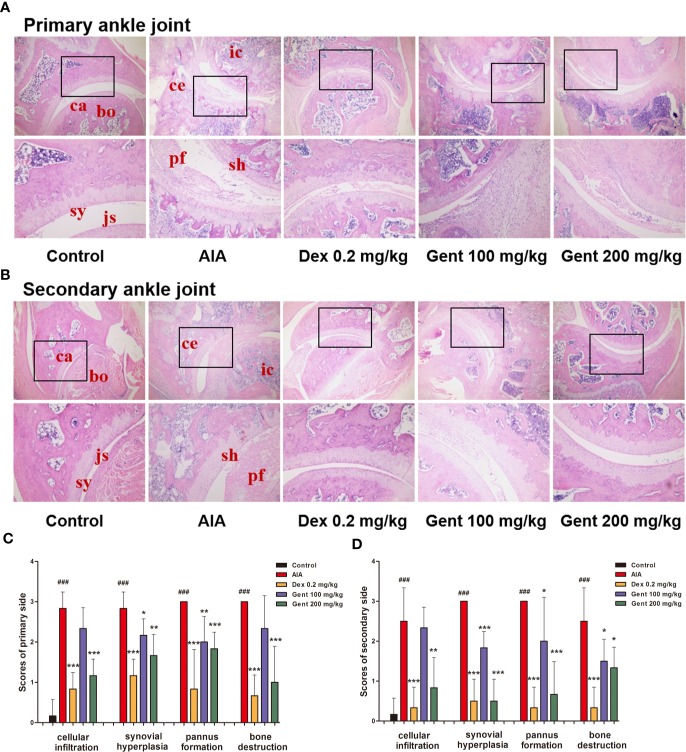
Delayed treatment with Gent reduces ankle joint damage in AIA model rats. **(A**, **B)** Damage to joint tissues was assessed *via* HE staining, with representative images being shown for all treatment groups. bo, bone; ca, cartilage; sy, synovium; js, joint space; sh, synovial hyperplasia; ce, cartilage erosion; ic, inflammatory cells; pf, pannus formation. Original magnification, 40 ×; high-power views, 100 ×. **(C, D)** A semi-quantitative histopathological score was assigned to both primary and secondary ankle joints based upon cellular infiltration, synovial hyperplasia, pannus formation, and bone destruction. Data were presented as mean ± SD (*n* = 6). ^###^*P* < 0.001 vs. control; *P < 0.05, **P < 0.01, and ***P < 0.001 vs. AIA group.

To further explore the anti-RA activity of Gent in this AIA model system, the joint histopathology of treated animals was assessed. Control rats exhibited normal joint architecture, whereas AIA model animals exhibited substantial inflammatory cell infiltration of the joints, pannus formation, synovial hyperplasia, and bone destruction (**Figures 3A, B**). Dex and Gent mediated dose-dependent reductions in inflammation, pannus formation, bone destruction, and synovial hyperplasia in treated animals (**Figures 3C, D**).

### The Impact of Gent on Body Weight in AIA Model Rats

**Figure 4:**
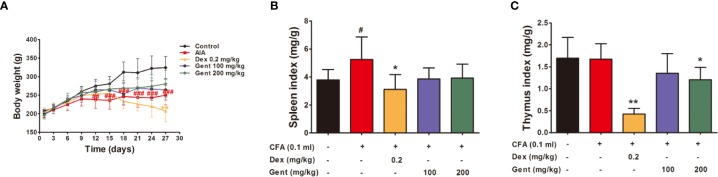
The impact of Gent on metabolism and immunity **(A)** Body weight in treated rats over time. **(B, C)** Spleen and thymus indices. Data were presented as mean ± SD (*n* = 10 for control group, 8 for AIA group, 9 for Dex 0.2 mg/kg group, 7 for Gent 100 mg/kg group, 7 for Gent 200 mg/kg group). ^#^*P* < 0.05, ^##^*P* < 0.01, and ^###^*P* < 0.001 vs. control; *P < 0.05, **P < 0.01 vs. AIA group.

Relative to control animals, AIA model rats exhibited significant reductions in body weight beginning on day 12 post-immunization owing to reductions in appetite. Relative to the AIA group, Dex-treated animals exhibited lower body weight on day 27 owing to differences in metabolic activity. However, Gent did not impact the body weight of treated animals (**Figure 4A**).

### The Impact of Gent on Immune Organ Index Values in AIA Model Rats

Spleen index values were significantly increased in AIA model animals at the end of the experimental period relative to control, consistent with the immunological basis for this disease. Relative to AIA model animals, Dex-treated rats exhibited significant reductions in immune organ index values consistent with impaired immunological functionality in treated animals. The Gent 200 mg/kg dose was also associated with a slight reduction in thymus index values, but it did not affect spleen index values (**Figures 4B, C**).

### Gent Treatment Alters NF-κB Signaling in the Joints of AIA Model Rats

**Figure 5:**
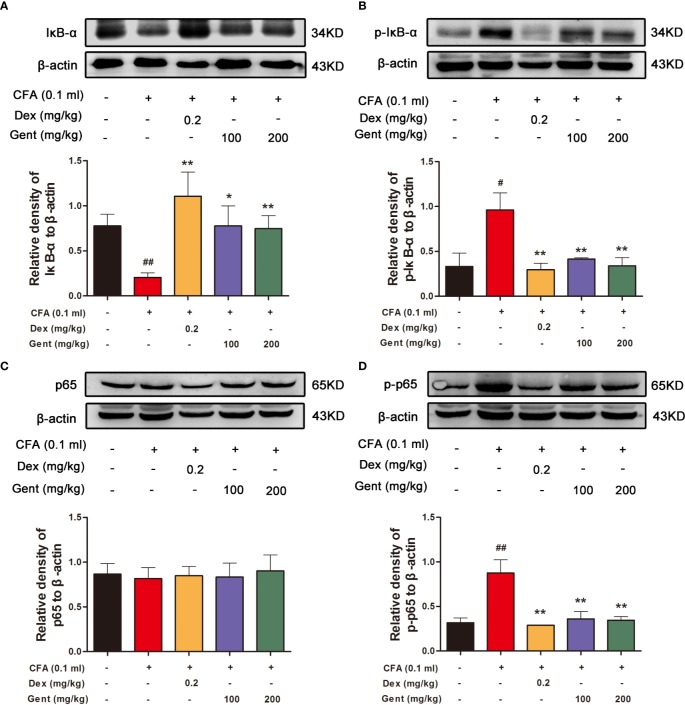
Gent alters NF-κB pathway activation in rat joint synovial tissue. Western blotting was used to measure IκBα **(A)**, p-IκBα **(B)**, p65 **(C)**, and p-p65 **(D)** expression in AIA rats. β-actin served as a normalization control. Data were presented as the mean ± SD from three independent experiments. ^#^*P* < 0.05, ^##^*P* < 0.01 vs. control; *P < 0.05, **P < 0.01 vs. AIA group.

To explore the mechanistic basis for Gent-mediated joint protection in AIA model rats, we next assessed synovial IκBα (**Figure 5A**), p-IκBα (**Figure 5B**), p65 (**Figure 5C**), and p-p65 (**Figure 5D**) expression in the synovial tissue of experimental animals *via* Western blotting. This analysis revealed that AIA model animals had significantly lower levels of synovial IκBα consistent with its degradation, and this was associated with concomitant increases in p-IκBα and p-p65 expression. In contrast, Gent and Dex markedly inhibited this IκBα degradation and reduced p-IκBα and p-p65 expression levels. This suggests that Gent may protect against joint damage in AIA model rats at least in part *via* suppressing the activation of NF-κB signaling.

### Primary RA-FLS Characterization

**Figure 6:**
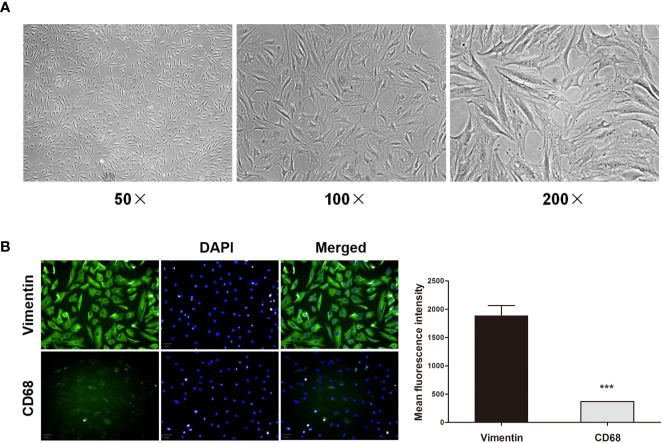
Primary RA-FLS characterization. **(A)** RA-FLS morphology was assessed by light microscopy (50 ×, 100 ×, and 200 ×). **(B)** Cells were stained for vimentin or CD68 (green) and with DAPI (blue), with FLS being vimentin-positive and CD68-negative (Scale bar: 50 μm). Data were presented as the mean ± SD from three independent experiments. ***P < 0.001 vs. vimentin group.

For *in vitro* experiments, FLS were initially characterized based upon their morphology and immunofluorescent staining profiles. These adherent cells were long fusiform or spindle-shaped morphological characteristics under light microscopy (**Figure 6A**), and were vimentin-positive and CD68-negative (**Figure 6B**). This confirmed that the purity of FLS was more than 90%, which was well-suited to use in downstream experiments.

### The Impact of Gent on RA-FLS Cell Viability

**Figure 7:**
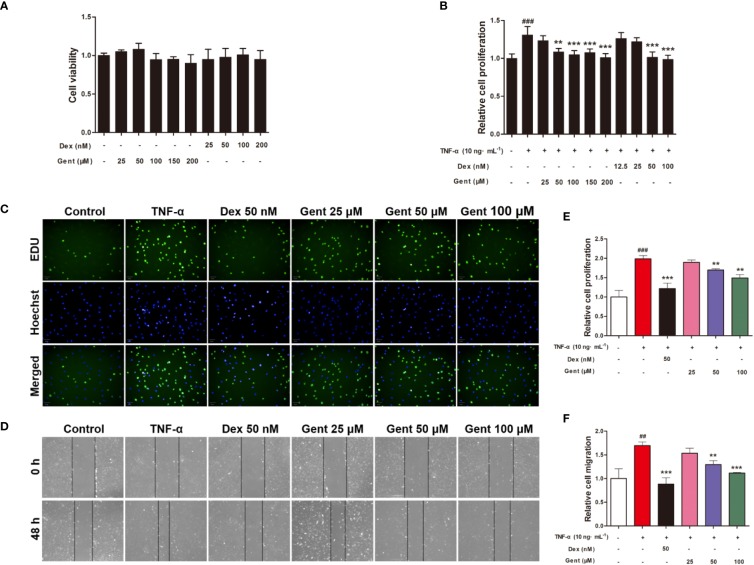
The impact of Gent on primary RA-FLS viability and TNF-α-induced proliferation and migration. **(A)** Cell viability was assessed *via* MTT assay after a 72-h treatment with the indicated Gent concentrations. **(B)** Following Gent or Dex pretreatment, RA-FLS were treated using 10 ng/mL TNF-α for 72 h, after which an MTT assay was used to quantify cell proliferation. **(C)** RA-FLS proliferation was also assessed based on EdU (green) incorporation, with DAPI-stained nuclei shown in blue (scale bar = 50 μm). **(D)** Cell migration in a wound healing assay was quantified following a 48-h incubation with or without the indicated Gent and Dex doses (100 × magnification). **(E, F)** Quantification of the data shown in C and D. Data were presented as the mean ± SD from three independent experiments. ^##^*P* < 0.01, ^###^*P* < 0.001 vs. control; **P < 0.01, ***P < 0.001 vs. TNF-α group.

We next assessed RA-FLS viability in response to a range of Gent concentrations. A subsequent MTT analysis revealed Gent and Dex to not induce significant cell mortality over a 72-h period at concentrations of up to 200 μM and 200 nM, respectively (**Figure 7A**).

### The Impact of Gent on TNF-α-Induced RA-FLS Proliferation

We next explored the ability of Gent to alter TNF-α-induced RA-FLS proliferation using MTT and EdU assays. TNF-α treatment was associated with a marked increase in RA-FLS proliferation, while Gent and Dex doses as low 50 μM and 50 nM, respectively, were able to inhibit this induced proliferation (**Figures 7B, C, E**). As a result, for downstream experiments Dex was used at a 50-nM concentration while Gent was used at 25-, 50-, and 100-μM concentrations.

### The Impact of Gent on TNF-α-Induced RA-FLS Migration

RA-FLS migration is a key mediator of RA pathology, driving the destruction of bone and cartilage tissue *in vivo*. To gauge the impact of Gent on these migratory processes, we conducted *in vitro* wound healing assays which revealed that a 48-h TNF-α treatment was sufficient to significantly enhance RA-FLS migration. Relative to TNF-α-treated cells, the migration of cells treated with Gent (50 μM or 100 μM) and Dex (50 nM) was significantly reduced in a dose-dependent fashion (**Figures 7D, F**).

### The Impact of Gent on TNF-α-Induced RA-FLS Proinflammatory Cytokine Production

**Figure 8:**
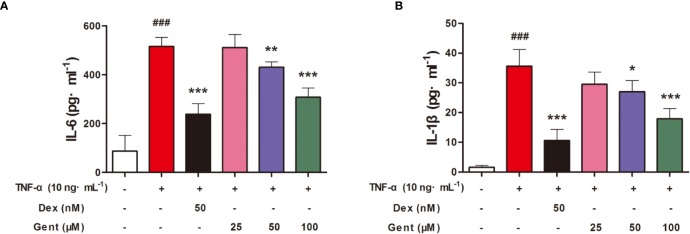
Gent suppresses RA-FLS proinflammatory cytokine production. Levels of IL-6 **(A)** and IL-1β **(B)** in cell supernatants were measured *via* ELISA. Data were presented as the mean ± SD from three independent experiments. ^###^*P* < 0.001 vs. control; *P < 0.05, **P < 0.01, and ***P < 0.001 vs. TNF-α group.

Next, we explored whether Gent was able to disrupt the production of proinflammatory cytokines by RA-FLS in response to TNF-α treatment. To that end, we measured supernatant IL-6 and IL-1β levels from treated cells, revealing that TNF-α stimulation resulted in a significant increase in both IL-6 (**Figure 8A**) and IL-1β (**Figure 8B**) production. Pre-treatment for 2 h with Gent (50 or 100 μM) and Dex (50 nM), however, significantly reduced the secretion of both of these inflammatory factors in a dose-dependent manner.

### The Impact of Gent on TNF-α-Induced ROS in RA-FLS

**Figure 9:**
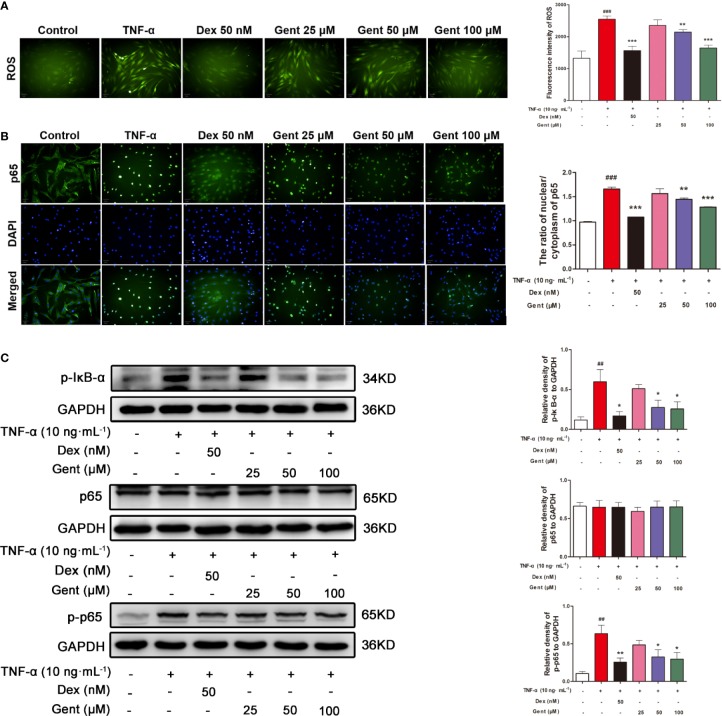
The impact of Gent on TNF-α-induced ROS production and NF-κB activity in RA-FLS. **(A)** DCFH-DA was used to detect intracellular ROS (green) (scale bar = 50 μm). **(B)** The translocation of p65 (green) into nuclei (blue) was assessed (scale bar = 50 μm). **(C)** p-IκBα, p65, and p-p65 levels in RA-FLS were measured *via* western blotting, with GAPDH as a loading control. Data were presented as the mean ± SD from three independent experiments. ^##^*P* < 0.01, ^###^*P* < 0.001 vs. control; *P < 0.05, **P < 0.01, and ***P < 0.001 vs. TNF-α group.

We next used the ROS-sensitive DCFH-DA dye to measure intracellular ROS levels in RA-FLS cells. We found that a 48-h treatment with TNF-α significantly enhanced these ROS levels, while pretreatment Gent (50 μM or 100 μM) and Dex (50 nM) was sufficient to suppress this ROS production (**Figure 9A**).

### The Impact of Gent on TNF-α-Induced NF-κB Signaling in RA-FLS

In order to assessed how Gent treatment impacted NF-κB signaling in RA-FLS cells, we next assessed p65 nuclear translocation *via* immunofluorescence microscopy. In control cells, p65 was distributed throughout the cytoplasm of these cells, whereas following a 20-min TNF-α stimulation it was localized almost exclusively in the nuclei of treated cells (**Figure 9B**). In cells pretreated with Gent (50 μM or 100 μM) and Dex, this TNF-α-induced nuclear translocation was significantly inhibited. Western blotting further confirmed that 48-h TNF-α treatment was associated with a significant upregulation of p-IκBα and p-p65 levels, whereas Gent (50 μM or 100 μM) and Dex pretreatment markedly reduced this upregulation (**Figure 9C**). Together, these findings suggest that Gent is capable of inhibiting the TNF-α-induced proliferation, migration, and inflammation of RA-FLS at least in part *via* inhibition of NF-κB signaling.

### The Impact of Gent on TNF-α-Induced NLRP3 Inflammasome Activation in RA-FLS

**Figure 10:**
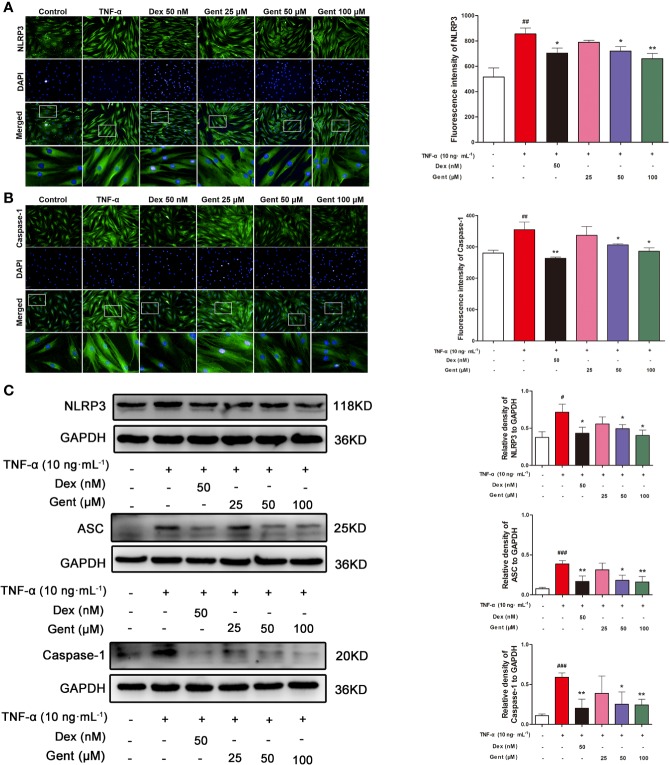
The impact of Gent on TNF-α-induced NLRP3 inflammasome activation in RA-FLS. **(A**, **B)** NLRP3 and caspase-1 levels in RA-FLS were assessed *via* immunofluorescence microscopy. Nuclei were stained with DAPI (blue) while NLRP3 and caspase-1 immunofluorescence were in green (scale bar = 50 µm). **(C)** NLRP3, ASC, and caspase-1 levels were assessed *via* Western blotting in RA-FLS, with GAPDH as a loading control. Data were presented as the mean ± SD from three independent experiments. ^#^*P* < 0.05, ^##^*P* < 0.01, and ^###^*P* < 0.001 vs. control; *P < 0.05, **P < 0.01 vs. TNF-α group.

Lastly, we assessed the ability of Gent treatment to modulate NLRP3 inflammasome activation in RA-FLS. We found that a 48-h treatment with TNF-α was sufficient to increase the expression of NLRP3, ASC, and caspase-1 at the protein level as measured *via* immunofluorescence microscopy or western blotting. In contrast, Gent (50 or 100 μM) and Dex were able to inhibit the upregulation of these three proteins, thus suppressing NLRP3 inflammasome activation (**Figures 10A–C**).

## Discussion

RA is a chronic progressive systemic autoimmune disease that can decrease the life expectancy of affected individuals by 3 to 20 years. The pathogenesis of RA is complicated, and as such treatment efforts typically aim to improve patient quality of life *via* slowing disease progression and relieving symptoms.

The *in vivo* study of RA is often conducted using AIA model animals as they exhibit immunological and histological features similar to those of human RA patients, thus allowing for the identification of novel RA treatment modalities (Wu et al., 2016). AIA development occurs in two stages. Initially, primary inflammation occurs within the immunized paw within 24 h of injection. During the secondary stage of this disease, which begins after ~14 days, secondary swelling of non-immunized paws begins to occur as a result of CFA-induced immune responses in model animals (Dekkers et al., 2017). In the present study, we found that Gent was able to relieve primary and secondary swelling as well as arthritis index scores in treated animals, thus suggesting that Gent can reduce arthritis progression in rats. The result was consistent with previous studies (Xie et al., 2019).

The sustained usage of high doses of Dex can lead to profound immunosuppression and a number of other side effects (Barnes and Adcock, 2009). The metabolic and immunomodulatory activity of different compounds is typically evaluated based on both body weight and immune organ index values in treated animals (Cao et al., 2019). We found that CFA treatment resulted in a reduction in rat body weight and increased spleen weight consistent with the stimulation of lymphocyte proliferation. Dex treatment led to significant decreases in both body weight and immune organ index values, indicating that it induced both significant immunosuppression and altered metabolic activity. Indeed, the adverse effects of Dex, such as affecting metabolism, immunosuppression, and infection, have limited it widely use in clinic (Dubois-Camacho et al., 2017). Gent 200 mg/kg reduced thymus index in AIA rats, but had no effect on spleen index and body weight. It revealed that Gent was relatively safe at the dose we chose.

FLS are specialized synovial cells that produce synovial fluid and serve as key regulators of join homeostasis. These cells are essential for the regulation of cartilage and joint damage, deformation, and destruction in RA (Kiener et al., 2010). FLS cells change dramatically during the course of RA progression, overcoming contact inhibition to undergo excessive tumor-like proliferation and migration that can drive local tissue damage and degradation (Bustamante et al., 2017). In this report, we found that Gent was able to suppress TNF-α-induced FLS migration and proliferation. Furthermore, *in vivo* we found that Gent treatment was able to reduce joint inflammatory cell infiltration, pannus formation, and bone destruction in AIA model animals, thus confirming that Gent is capable of suppressing pathogenic inflammation in the context of RA progression.

NF-κB signaling is a central regulator of inflammation. At baseline, NF-κB is sequestered in the cytoplasm *via* its interaction with IκB proteins, thus inhibiting its activation. In response to certain stimuli, however, these IκB proteins are phosphorylated and subsequently degraded, allowing NF-κB to undergo translocation into the nucleus wherein it can upregulate a number of different pro-inflammatory cytokines (Bianco et al., 2019; Oh et al., 2019). In this study, we confirmed that NF-κB signal pathway activation occurred in our model system and that Gent was able to suppress IκBα degradation and associated increases in p-IκBα and p-p65 levels *in vivo* in the joints of AIA model rats. This was additionally confirmed in TNF-α-stimulated RA-FLS cells *in vitro*, with Gent significantly inhibiting p65 nuclear translocation and decreasing p-IκBα and p-p65 levels in treated cells.

IL-6 and IL-1β are inflammatory cytokines that can be induced in a number of contexts whereupon they can drive innate and adaptive immunity. The unrestricted secretion of IL-6 and IL-1β is thought to be linked to the pathogenesis of numerous conditions including RA, highlighting the importance of carefully regulating the production of these inflammatory factors *in vivo* in order to ensure normal tissue homeostasis and immunological functionality (Ogura et al., 2008; Joosten et al., 2013). The result of our study was consistent with the previous finding that Gent treatment was able to significantly reduce IL-6 and IL-1β secretion by TNF-α-stimulated RA-FLS (Zhang et al., 2019). However, it has not been reported whether the effect of Gent on RA-FLS inflammatory factors is related to the ROS-NF-κB-NLRP3 inflammasome axis.

Furthermore, our study further clarified the mechanism by which Gent regulated abnormal proliferation, migration, and inflammatory processes of RA-FLS. NLRP3 inflammasome assembly results in caspase-1 activation and consequent IL-1β secretion (Gaidt et al., 2016; Liu et al., 2019). The NLRP3 has been found to serve as a key driver of several autoimmune conditions including both RA and systemic lupus erythematosus (SLE) (Shen et al., 2018). ROS are signaling intermediates that can drive both NLRP3 inflammasome and NF-κB activation (Wu et al., 2018; Zhao et al., 2019). In the present study, we found that TNF-α treatment drove a significant increase in ROS levels in RA-FLS as well as significant upregulation of NLRP3/ASC/pro-caspase-1, and caspase-1 in these cells, consistent with NLRP3 inflammasome activation. Gent pretreatment, however, suppressed these TNF-α-induced changes. NLRP3 inflammasome activation is a two-step procedure, with both priming and activation being necessary. In our model system, the ROS-NF-κB pathway was able to prime the NLRP3 inflammasome, inducing pro-IL-1β synthesis and NLRP3/ASC/pro-caspase-1 upregulation. Stimulation then triggered the activation of this primed NLRP3 inflammasome, resulting in caspase-1 activation. Once activated, caspase-1 cleaves pro-IL-1β into mature IL-1β. Together, our findings ultimately suggest that Gent is able to suppress NLRP3 inflammasome activation, and this suppression is associated with inhibition of ROS production and NF-κB activation.

## Conclusion

In summary, the results of this study provide clear evidence that Gentiopicroside can protect against the progression of RA. This anti-RA efficacy is at least partially attributable to Gentiopicroside-mediated inhibition of ROS-NF-κB-induced NLRP3 inflammasome activation. Gentiopicroside may therefore have novel value as a therapeutic agent that can treat RA *via* inhibiting this ROS-NF-κB-NLRP3 inflammasome axis, although further clinical trials will be needed to validate this possibility.

## Data Availability Statement

All datasets generated for this study are included in the article/**Supplementary Material**.

## Ethics Statement

The animal study was reviewed and approved by The Animal Ethics Committee of the Yantai University.

## Author Contributions

MW, WX, LZ, and FF conceived and designed the study. HL provided synovial tissues. MW, YW, YHa, XW, YHu, and YL assisted in carrying out the experiments. MW, HL, and YD wrote the manuscript. LZ and FF helped to proofread the article. All authors contributed to analysis and interpretation of the data and approved the final manuscript.

## Funding

This study was supported by the Key R&D Program Projects in Yantai City (2019XDHZ109), the National Science Foundation of China (No. 81803546, No. 81973547), and the Natural Science Foundation of Shandong Province (No. ZR2018LH024, No. ZR2017MH068).

## Conflict of Interest

The authors declare that the research was conducted in the absence of any commercial or financial relationships that could be construed as a potential conflict of interest.

## Abbreviations

RA, rheumatoid arthritis; AIA, adjuvant-induced arthritis; FLS, fibroblast-like synoviocytes; Gent, Gentiopicroside; Dex, dexamethasone; TNF-α, tumor necrosis factor alpha; NSAIDs, non-steroidal anti-inflammatory drugs; H&E, Harris hematoxylin; CMC-Na, sodium carboxyl methyl cellulose; MTT, 3-(4,5-dimethylthiazol-2-yl)-2,5-dimethyltetrazolium bromide; ROS, reactive oxygen species; NF-κB, nuclear factor kappa B; NLRP3, NOD-like receptor protein 3; ASC, speck-like protein containing a CARD.
