# Supplementary material for: Anti-Rheumatic Properties of Gentiopicroside Are Associated With Suppression of ROS-NF-κB-NLRP3 Axis in Fibroblast-Like Synoviocytes and NF-κB Pathway in Adjuvant-Induced Arthritis
Source: Front Pharmacol. 2020 May 4;11:515. doi: 10.3389/fphar.2020.00515 (PMC7232611; doi:10.3389/fphar.2020.00515)

The original western blots of each figure.

Figure 5A

IκBα: Control, AIA, AIA+Dex(0.2mg/kg), AIA+Gent(100mg/kg), AIA+Gent(200mg/kg)

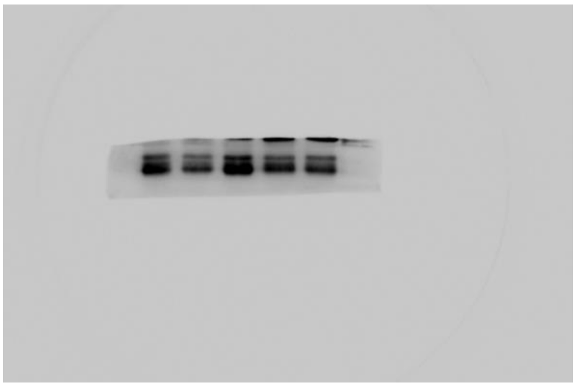

β-actin: Control, AIA, AIA+Dex(0.2mg/kg), AIA+Gent(100mg/kg), AIA+Gent(200mg/kg)

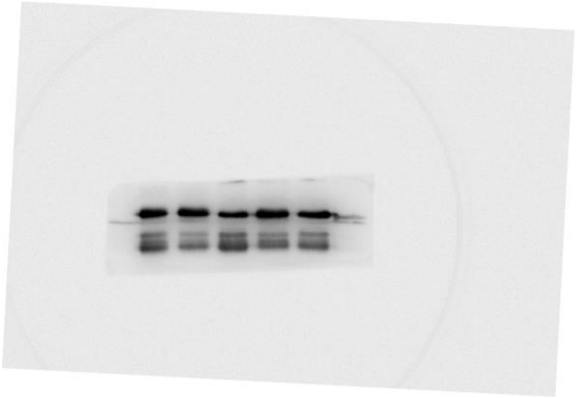

Figure 5B

p-IκBα: Control, AIA, AIA+Dex(0.2mg/kg), AIA+Gent(100mg/kg), AIA+Gent(200mg/kg)

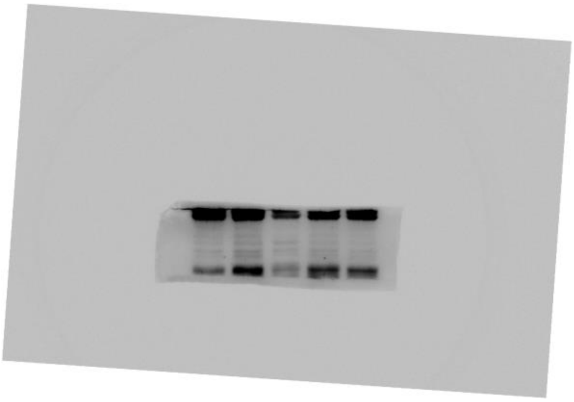

β-actin: Control, AIA, AIA+Dex(0.2mg/kg), AIA+Gent(100mg/kg), AIA+Gent(200mg/kg)

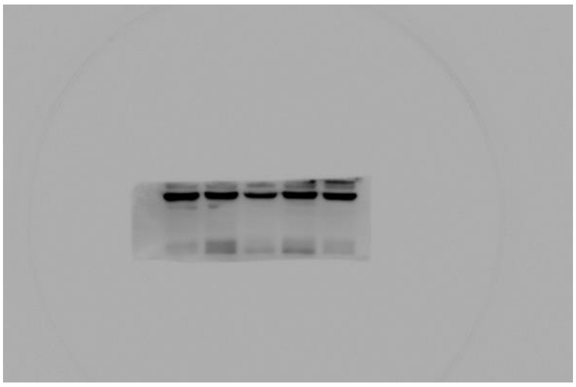

Figure 5C

p65: Control, AIA, AIA+Dex(0.2mg/kg), AIA+Gent(100mg/kg), AIA+Gent(200mg/kg)

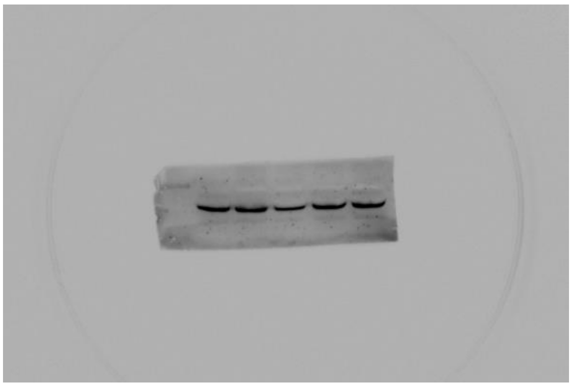

β-actin: Control, AIA, AIA+Dex(0.2mg/kg), AIA+Gent(100mg/kg),

AIA+Gent(200mg/kg)

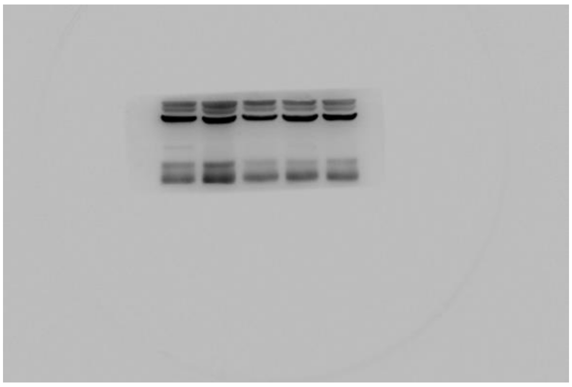

Figure 5D

p-p65: Control, AIA, AIA+Dex(0.2mg/kg), AIA+Gent(100mg/kg), AIA+Gent(200mg/kg)

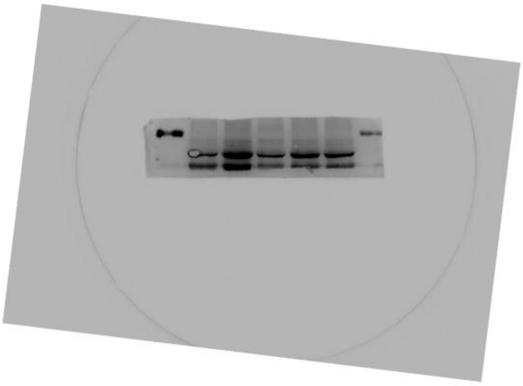

β-actin: Control, AIA, AIA+Dex(0.2mg/kg), AIA+Gent(100mg/kg), AIA+Gent(200mg/kg)

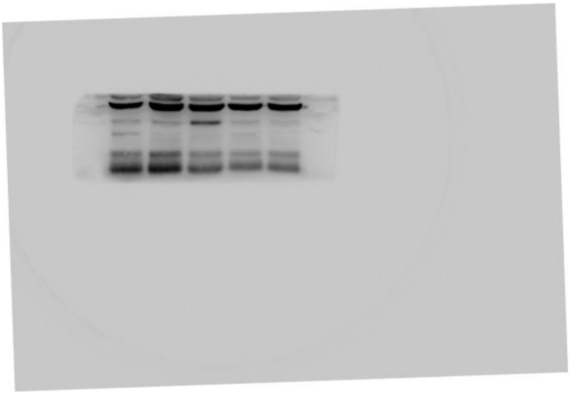

Figure 9C

p-IκBα: Control, TNF-α, TNF-α+Dex(50nM), TNF-α+Gent(25μM), TNF-α+Gent(50μM), TNF-α+Gent(100μM)

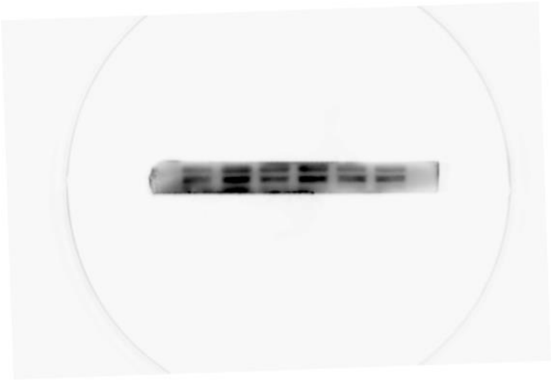

GAPDH: Control, TNF-α, TNF-α+Dex(50nM), TNF-α+Gent(25μM), TNF-α+Gent(50μM), TNF-α+Gent(100μM)

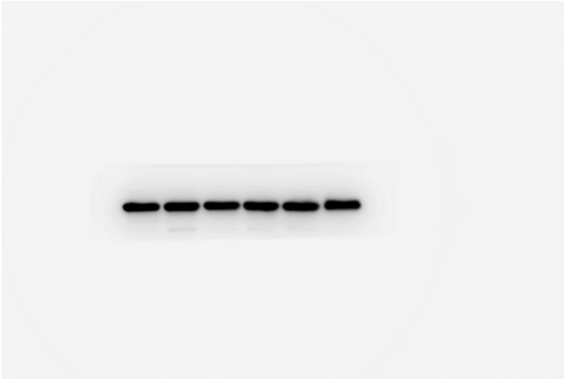

p65: Control, TNF-α, TNF-α+Dex(50nM), TNF-α+Gent(25μM), TNF-α+Gent(50μM), TNF-α+Gent(100μM)

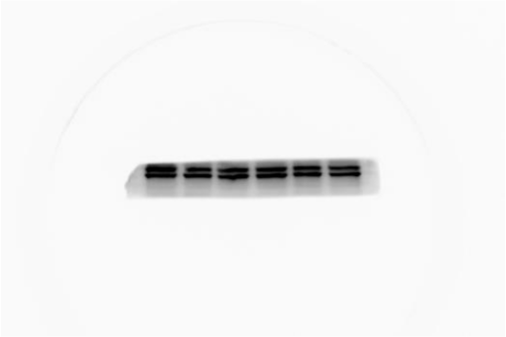

GAPDH: Control, TNF-α, TNF-α+Dex(50nM), TNF-α+Gent(25μM), TNF-

α+Gent(50μM), TNF-α+Gent(100μM)

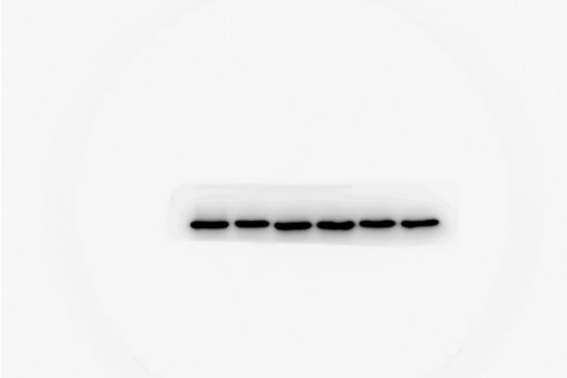

p-p65: Control, TNF-α, TNF-α+Dex(50nM), TNF-α+Gent(25μM), TNF-α+Gent(50μM), TNF-α+Gent(100μM)

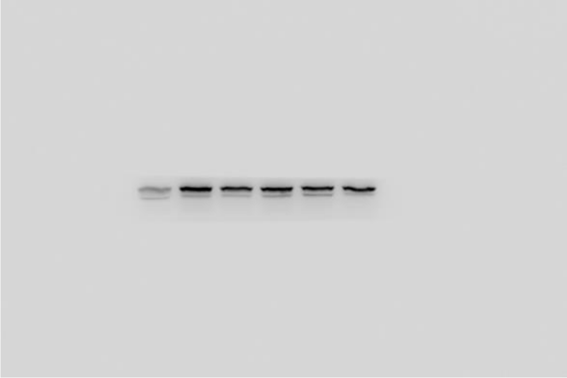

GAPDH: Control, TNF-α, TNF-α+Dex(50nM), TNF-α+Gent(25μM), TNF-α+Gent(50μM), TNF-α+Gent(100μM)

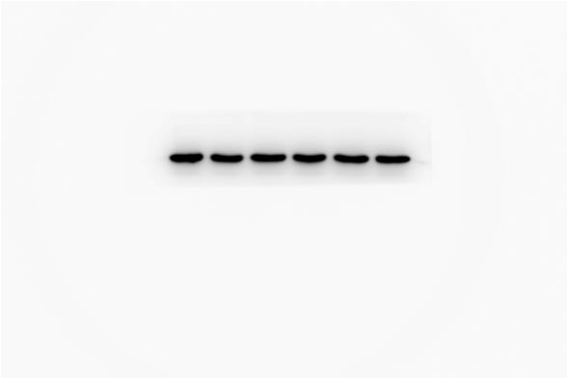

Figure 10C

NLRP3: Control, TNF-α, TNF-α+Dex(50nM), TNF-α+Gent(25μM), TNF-α+Gent(50μM), TNF-α+Gent(100μM)

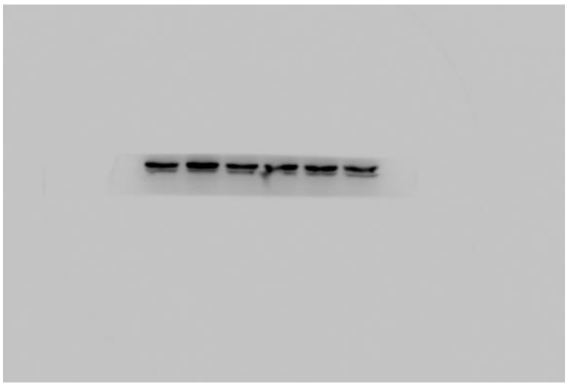

GAPDH: Control, TNF-α, TNF-α+Dex(50nM), TNF-α+Gent(25μM), TNF-α+Gent(50μM), TNF-α+Gent(100μM)

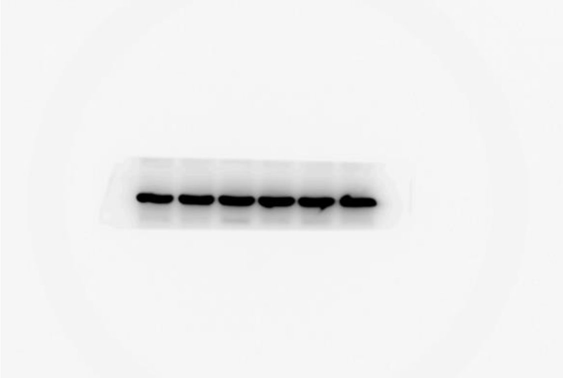

ASC: Control, TNF-α, TNF-α+Dex(50nM), TNF-α+Gent(25μM), TNF-α+Gent(50μM), TNF-α+Gent(100μM)

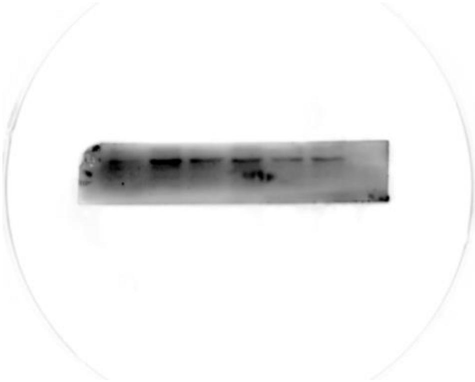

GAPDH: Control, TNF-α, TNF-α+Dex(50nM), TNF-α+Gent(25μM), TNF-α+Gent(50μM), TNF-α+Gent(100μM)

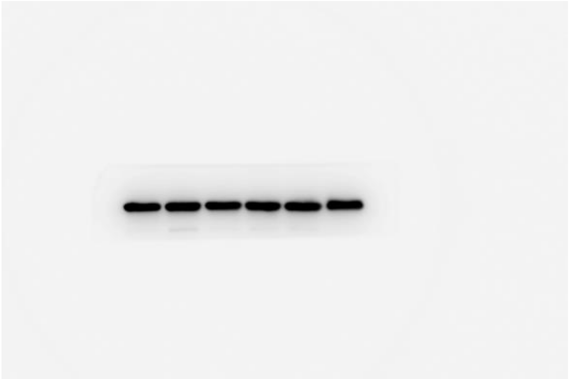

Caspase-1: Control, TNF-α, TNF-α+Dex(50nM), TNF-α+Gent(25μM), TNF-α+Gent(50μM), TNF-α+Gent(100μM)

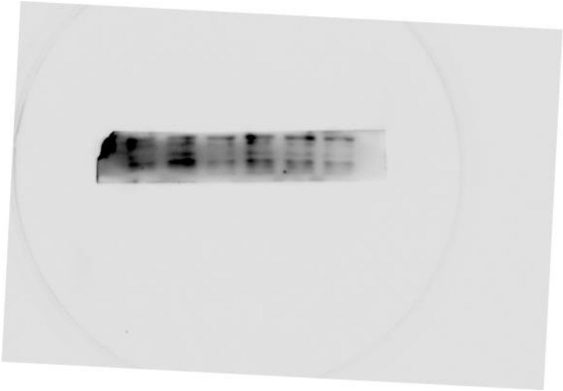

GAPDH: Control, TNF-α, TNF-α+Dex(50nM), TNF-α+Gent(25μM), TNF-α+Gent(50μM), TNF-α+Gent(100μM)

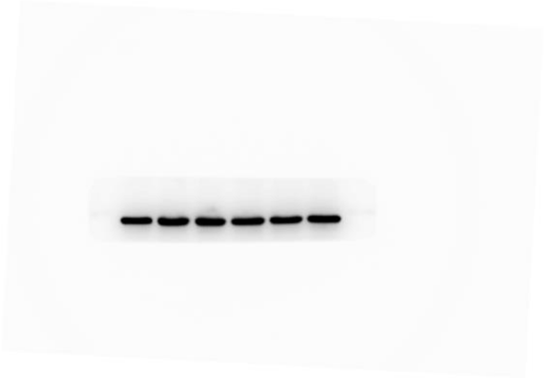

Supplement: Supplementary file 2 [file DataSheet_2.pdf]
