# Supplementary material for: Anti-Rheumatic Properties of Gentiopicroside Are Associated With Suppression of ROS-NF-κB-NLRP3 Axis in Fibroblast-Like Synoviocytes and NF-κB Pathway in Adjuvant-Induced Arthritis
Source: Front Pharmacol. 2020 May 4;11:515. doi: 10.3389/fphar.2020.00515 (PMC7232611; doi:10.3389/fphar.2020.00515)

Figure 3A: The original figures of primary ankle joint

Control:

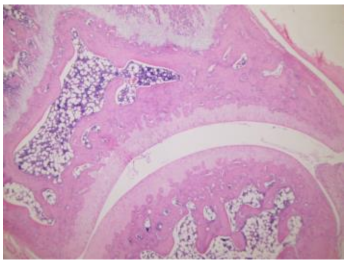

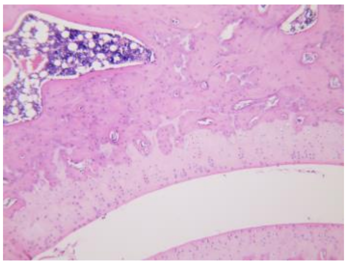

AIA:

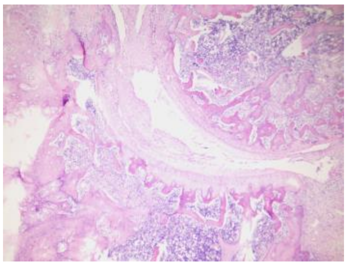

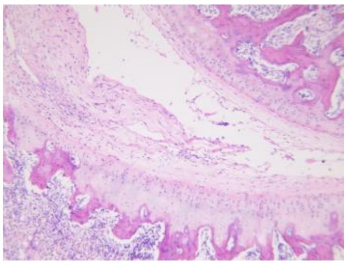

Dex 0.2 mg/kg:

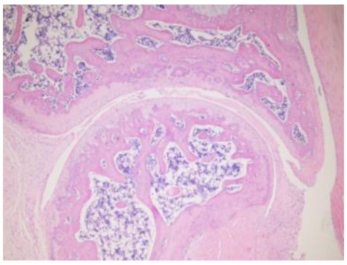

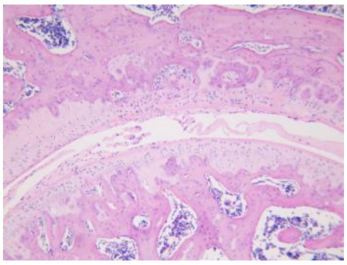

Gent 100 mg/kg:

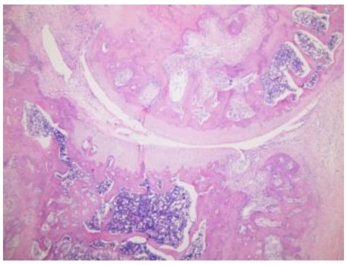

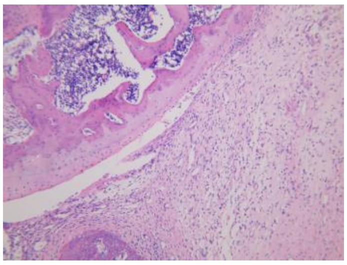

Gent 200 mg/kg:

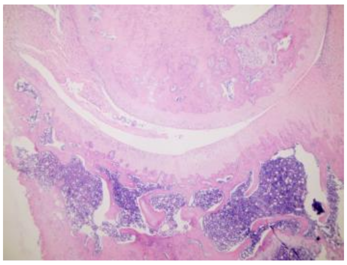

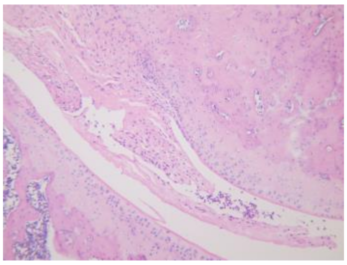

Figure 3B: The original figures of secondary ankle joint

Control:

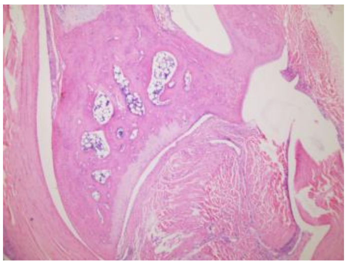

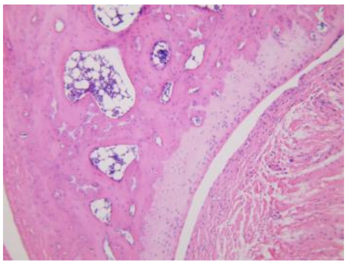

AIA:

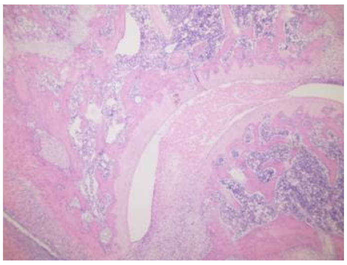

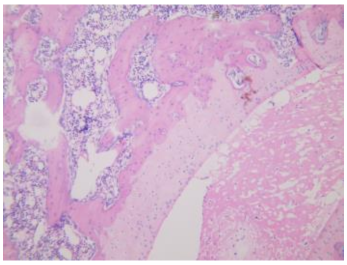

Dex 0.2 mg/kg:

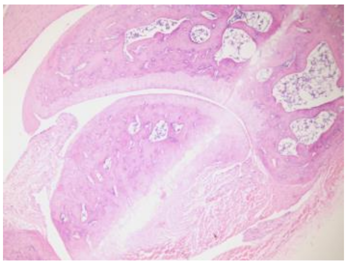

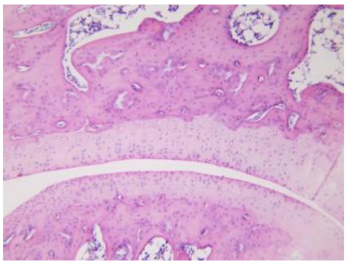

Gent 100 mg/kg:

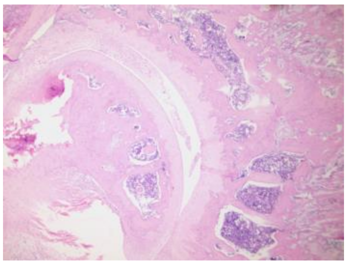

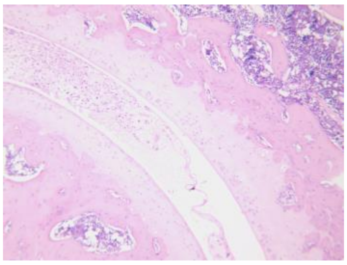

Gent 200 mg/kg:

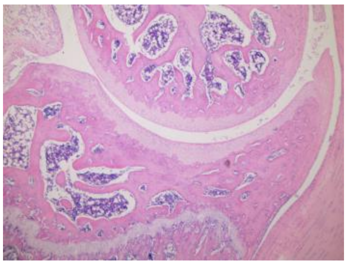

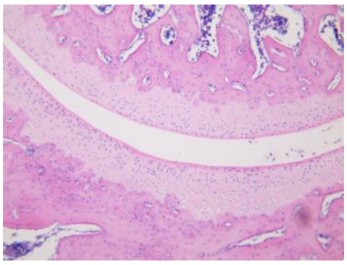

Figure 5: The original western blots of Figure 5

Figure 5A

IκBα: Control, AIA, Dex 0.2 mg/kg, Gent 100 mg/kg, Gent 200 mg/kg

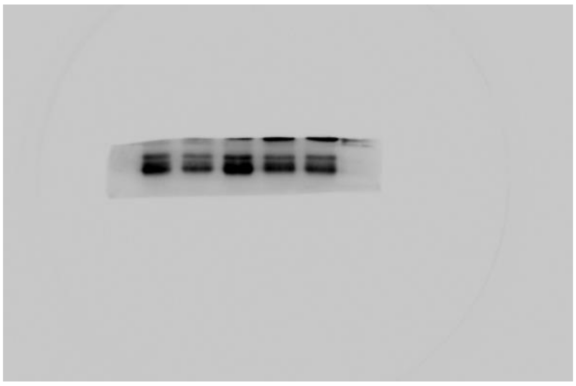

β-actin: Control, AIA, Dex 0.2 mg/kg, Gent 100 mg/kg, Gent 200 mg/kg

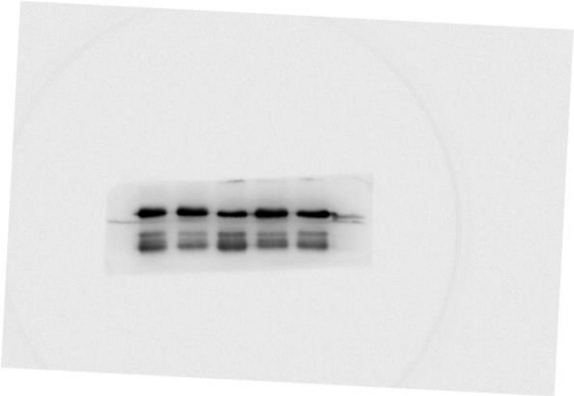

Figure 5B

p-IκBα: Control, AIA, Dex 0.2 mg/kg, Gent 100 mg/kg, Gent 200 mg/kg

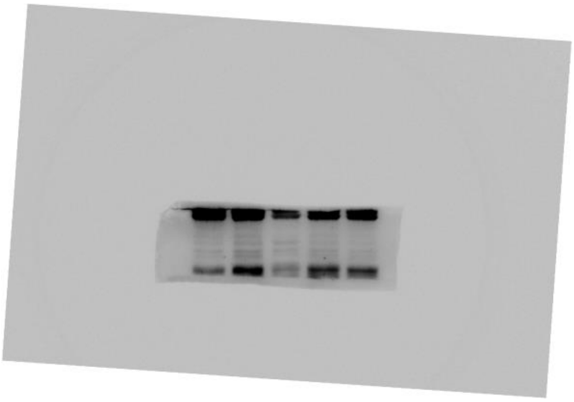

β-actin: Control, AIA, Dex 0.2 mg/kg, Gent 100 mg/kg, Gent 200 mg/kg

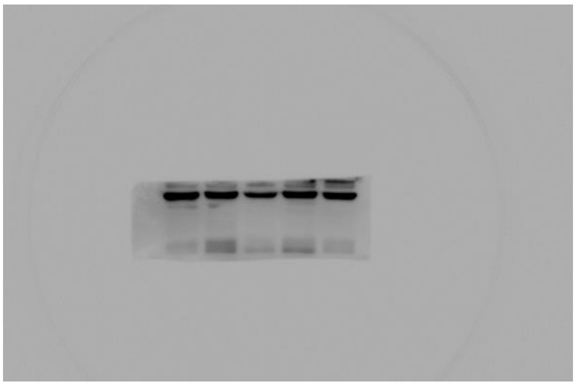

Figure 5C

p65: Control, AIA, Dex 0.2 mg/kg, Gent 100 mg/kg, Gent 200 mg/kg

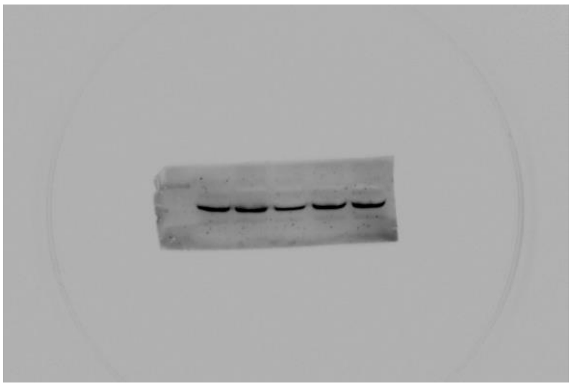

β-actin: Control, AIA, Dex 0.2 mg/kg, Gent 100 mg/kg, Gent 200 mg/kg

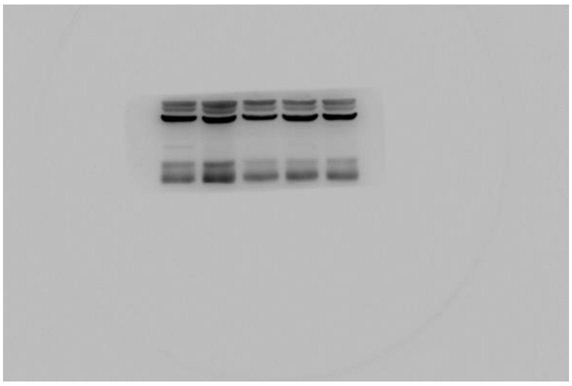

Figure 5D

p-p65: Control, AIA, Dex 0.2 mg/kg, Gent 100 mg/kg, Gent 200 mg/kg

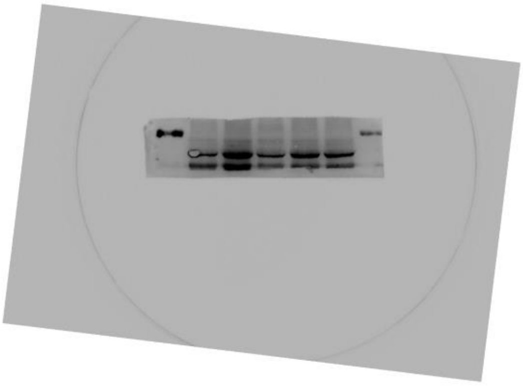

β-actin: Control, AIA, Dex 0.2 mg/kg, Gent 100 mg/kg, Gent 200 mg/kg

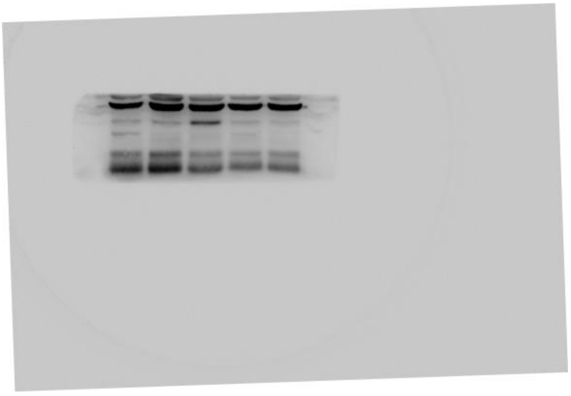

Figure 6A: The original figures of RA-FLS morphology

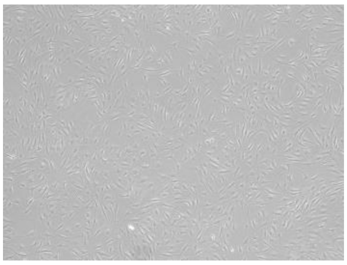

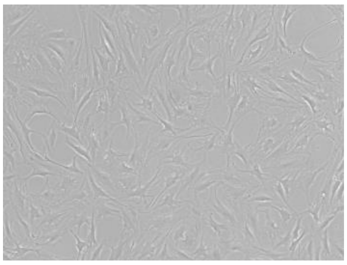

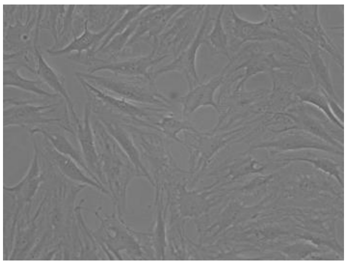

Figure 6B: The original figures of immunofluorescent staining

Vimentin:

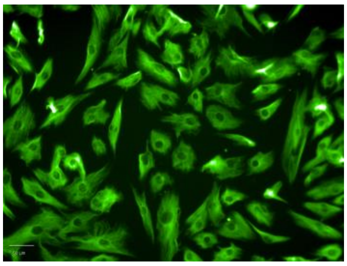

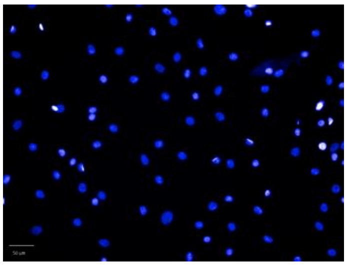

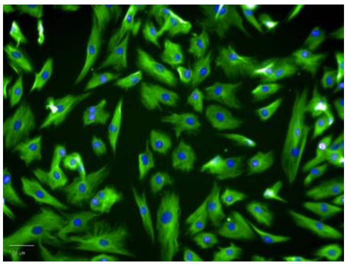

CD68:

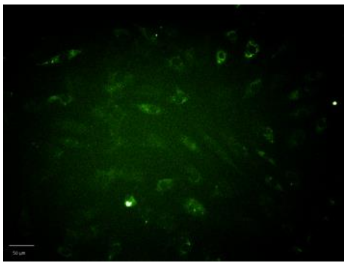

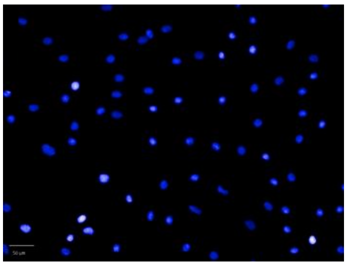

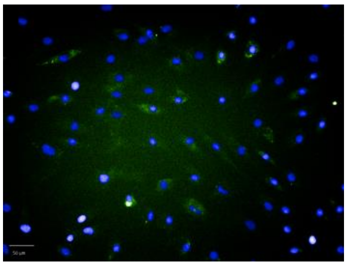

Figure 7C: The original figures of RA-FLS proliferation

Control:

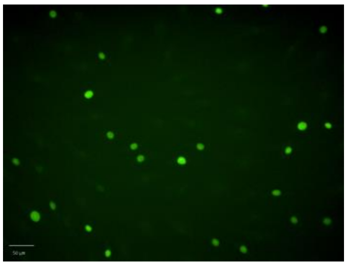

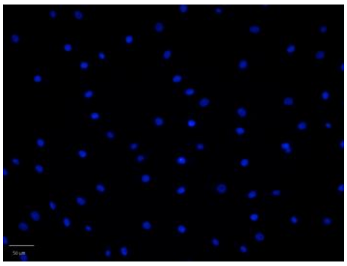

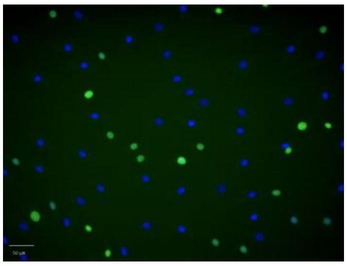

TNF-α:

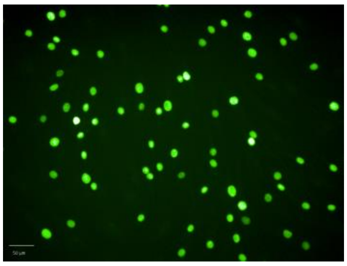

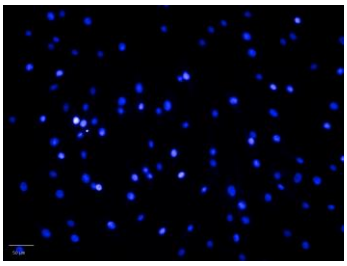

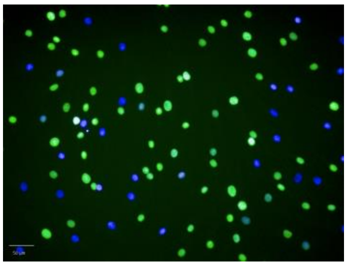

Dex 50 nM:

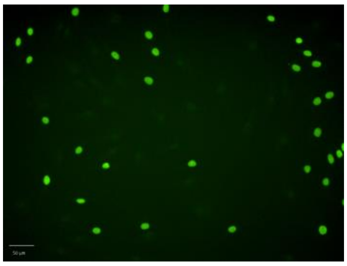

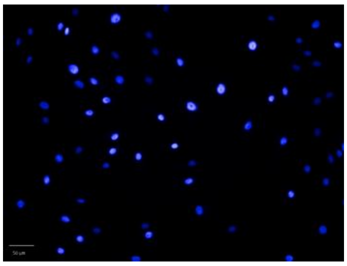

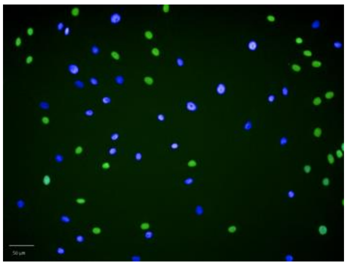

Gent 25 µM:

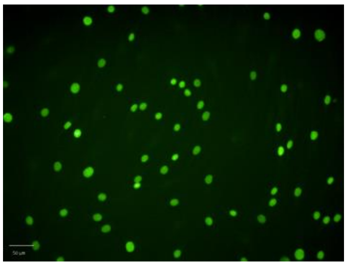

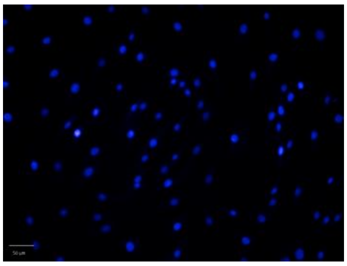

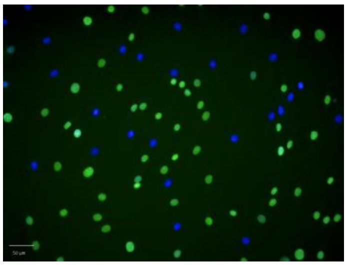

Gent 50µM:

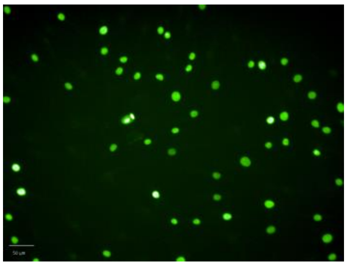

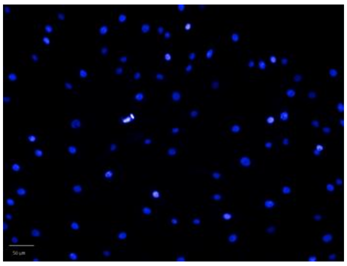

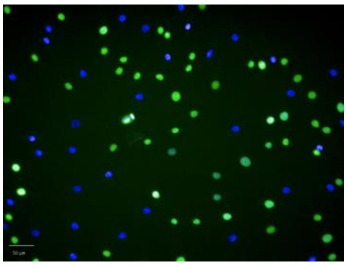

Gent 100 μM:

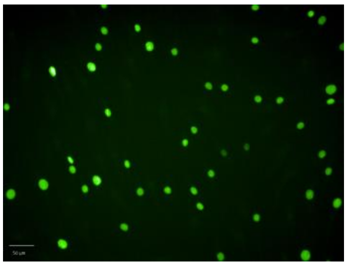

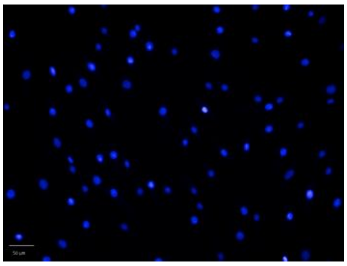

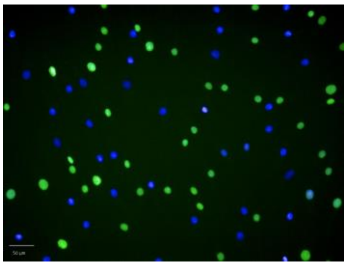

Figure 7D: The original figures of RA-FLS migration

Control:

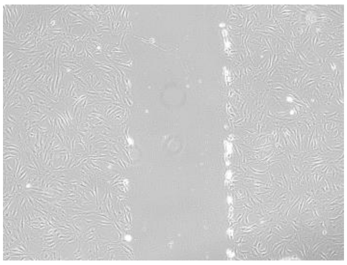

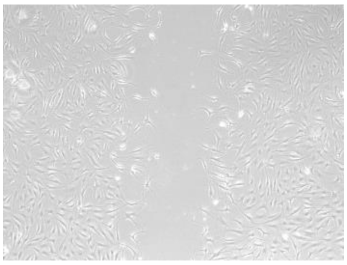

TNF-α:

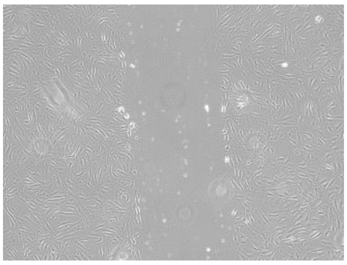

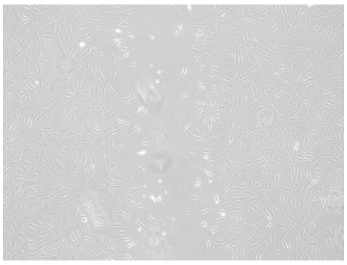

Dex 50 nM:

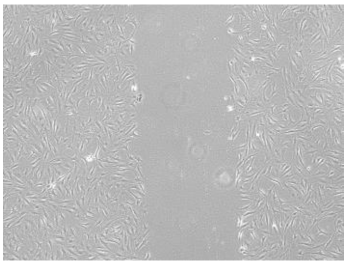

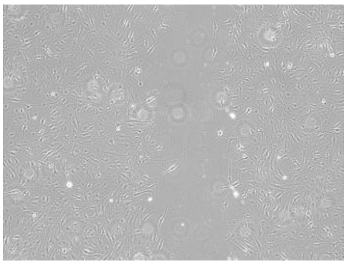

Gent 25 μM:

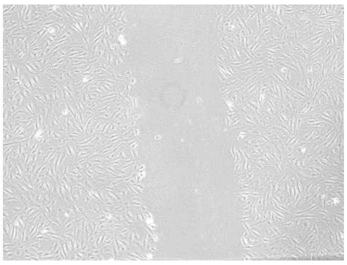

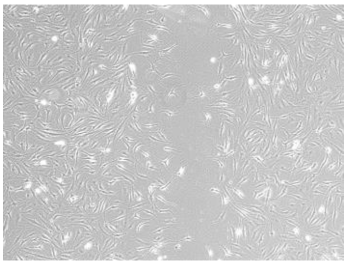

Gent 50μM:

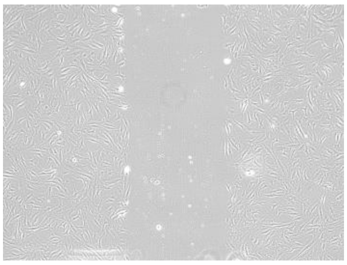

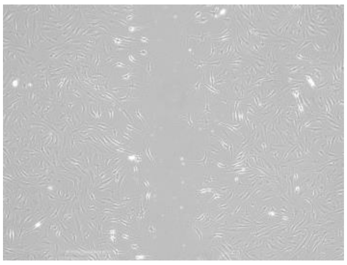

Gent 100 μM:

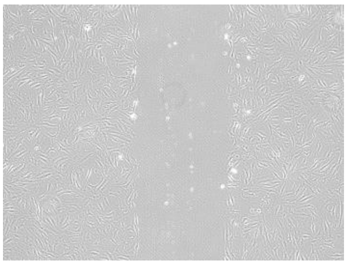

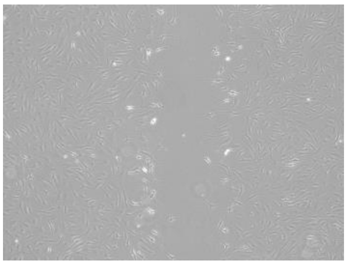

Figure 9A: The original figures of ROS

Control:

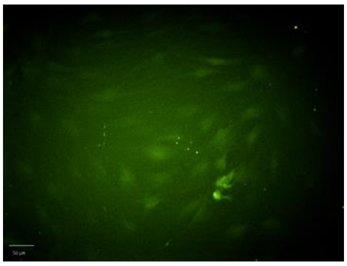

TNF-α:

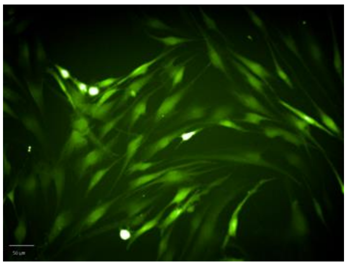

Dex 50 nM:

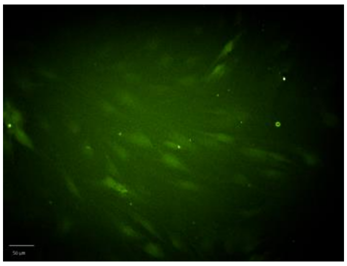

Gent 25 μM:

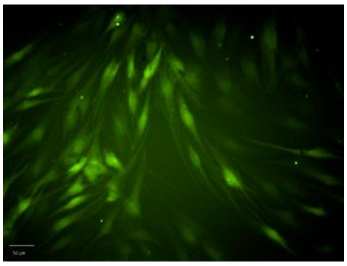

Gent 50 μM:

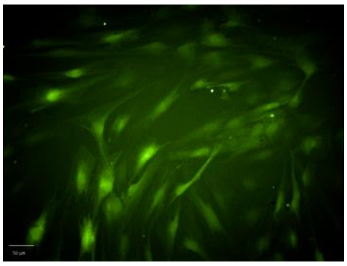

Gent 100 μM:

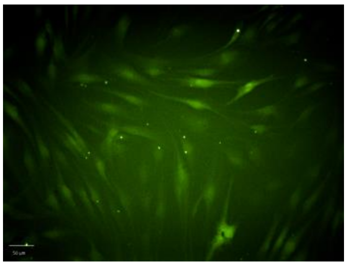

Figure 9B: The original figures of p65 nuclear translocation

Control:

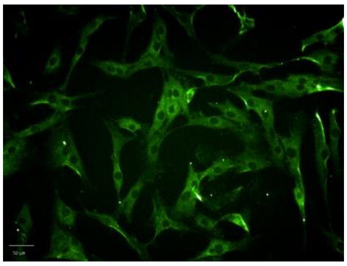

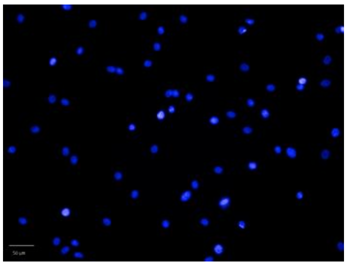

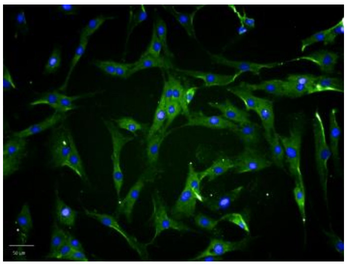

TNF-α:

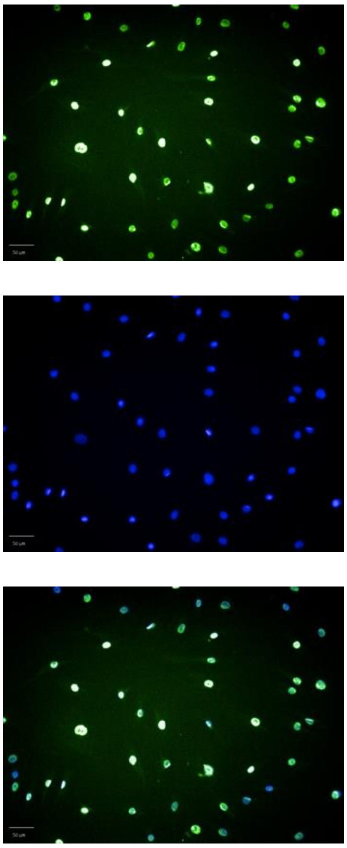

Dex 50 nM:

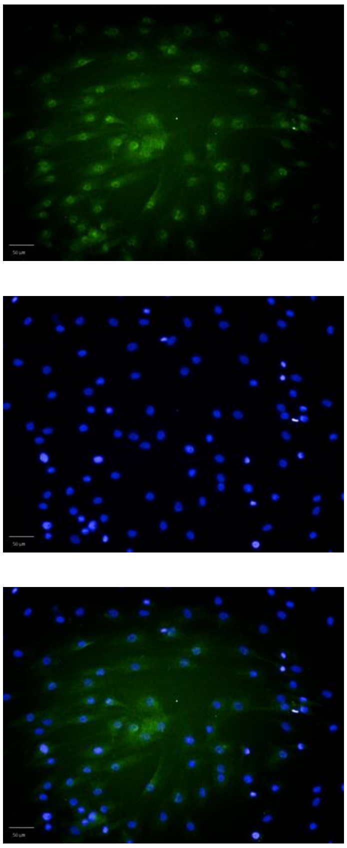

Gent 25 μM:

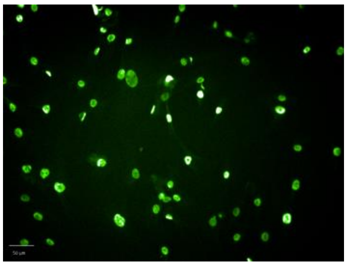

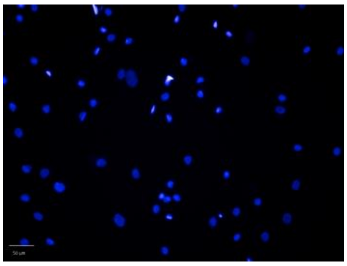

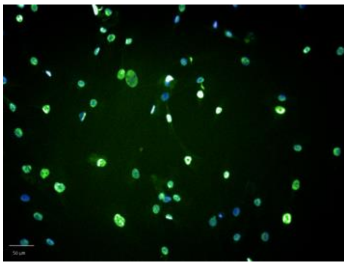

Gent 50 μM:

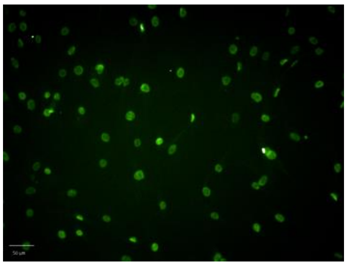

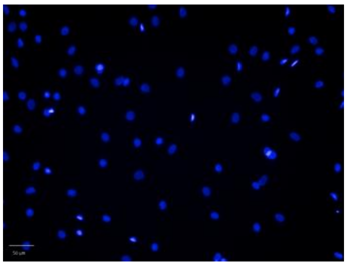

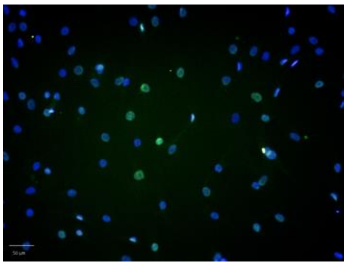

Gent 100 μM:

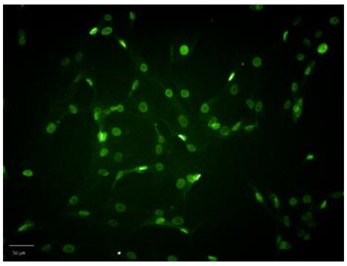

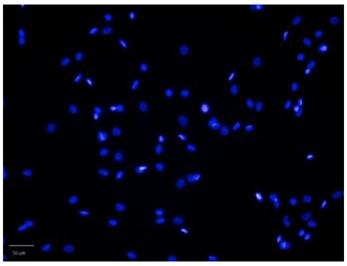

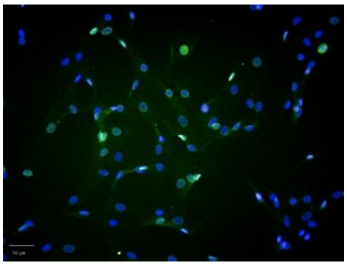

Figure 9C: The original western blots of Figure 9C

p-IκBα: Control, TNF-α, Dex 50 nM, Gent 25 μM, Gent 50 μM, Gent 100 μM

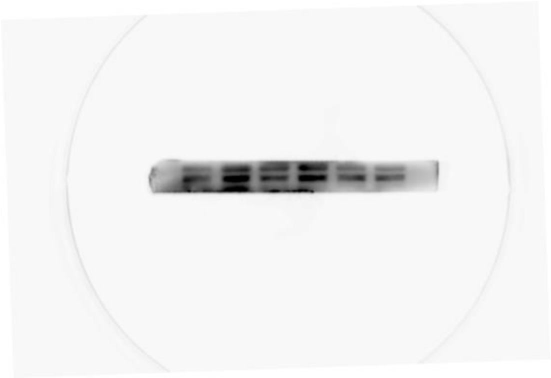

GAPDH: Control, TNF-α, Dex 50 nM, Gent 25 μM, Gent 50 μM, Gent 100 μM

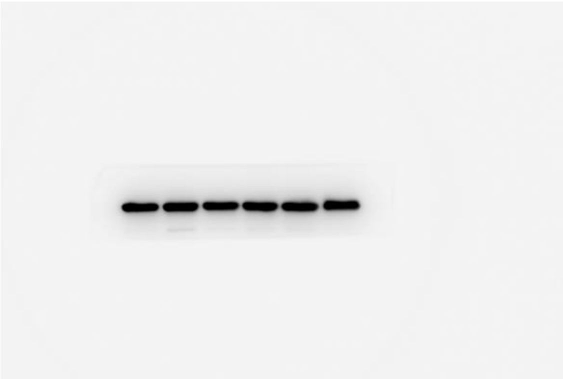

p65: Control, TNF-α, Dex 50 nM, Gent 25 μM, Gent 50 μM, Gent 100 μM

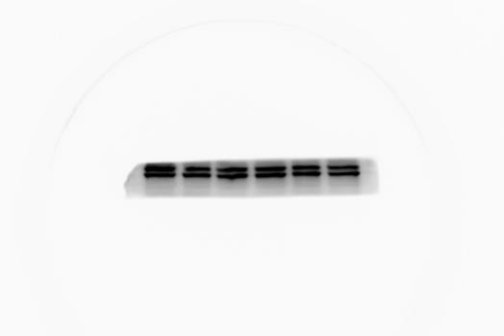

GAPDH: Control, TNF-α, Dex 50 nM, Gent 25 μM, Gent 50 μM, Gent 100 μM

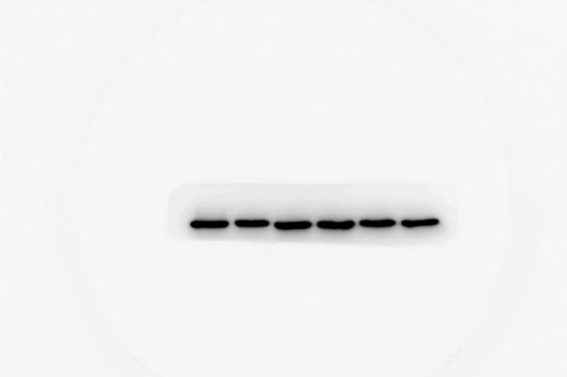

p-p65: Control, TNF-α, Dex 50 nM, Gent 25 μM, Gent 50 μM, Gent 100 μM

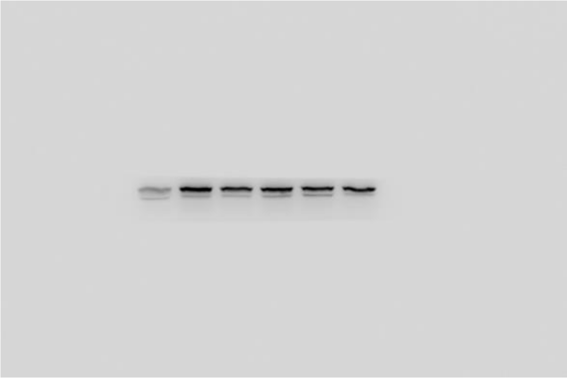

GAPDH: Control, TNF-α, Dex 50 nM, Gent 25 μM, Gent 50 μM, Gent 100 μM

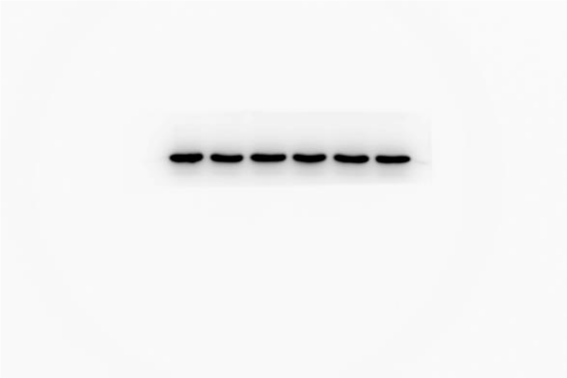

Figure 10A: The original figures of NLRP3

Control:

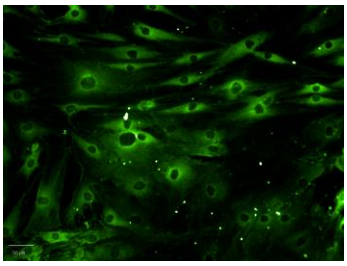

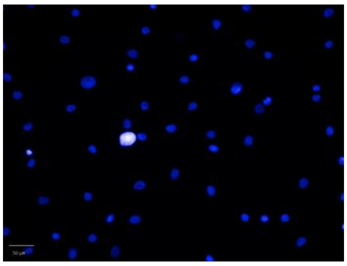

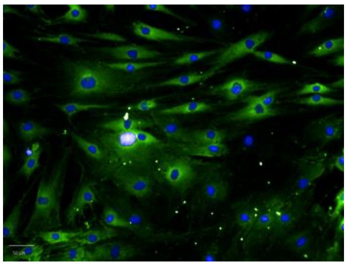

TNF-α:

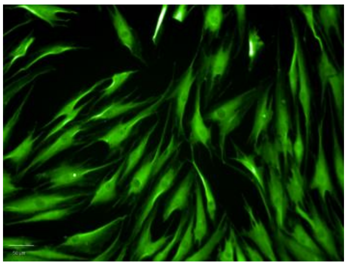

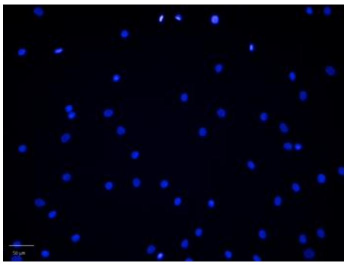

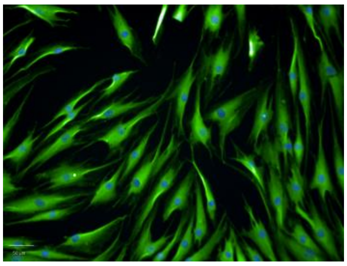

Dex 50 nM:

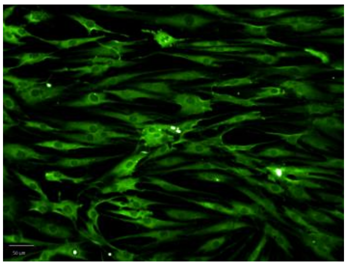

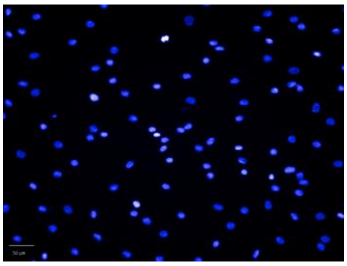

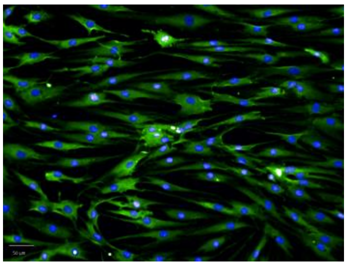

Gent 25 μM:

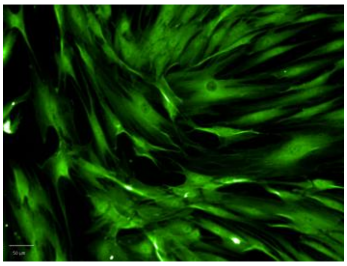

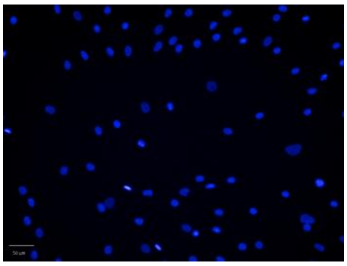

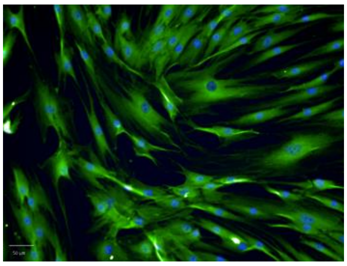

Gent 50 μM:

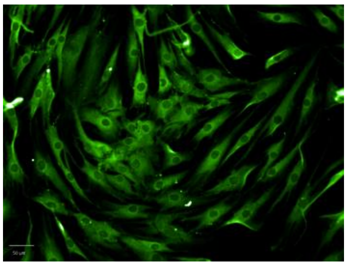

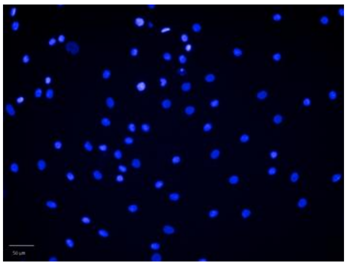

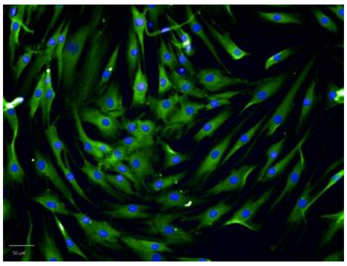

Gent 100 μM:

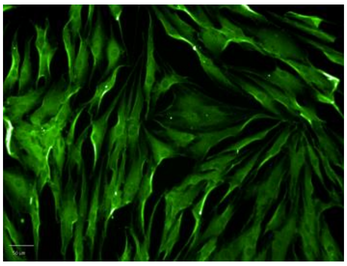

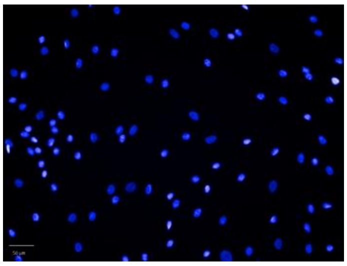

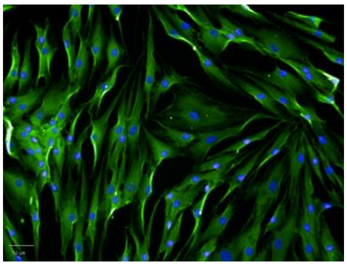

Figure 10B: The original figures of caspase-1

Control:

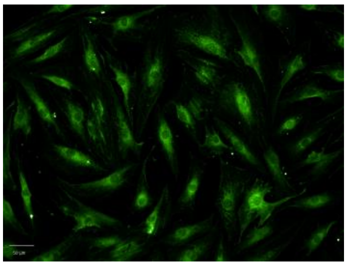

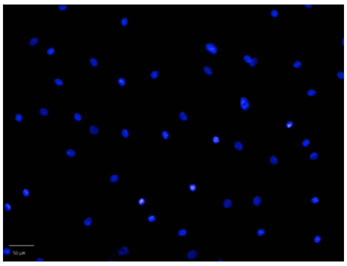

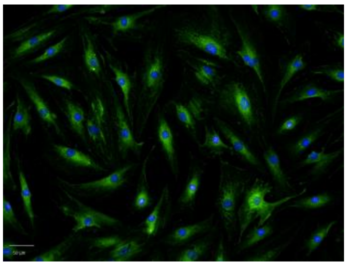

TNF-α:

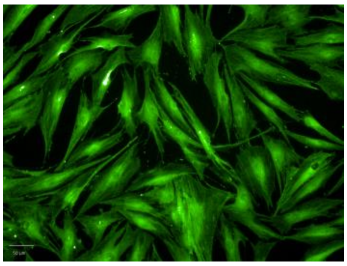

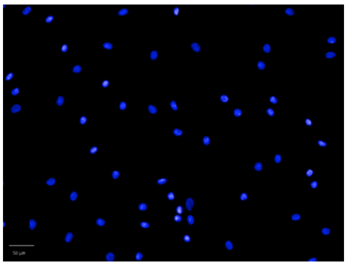

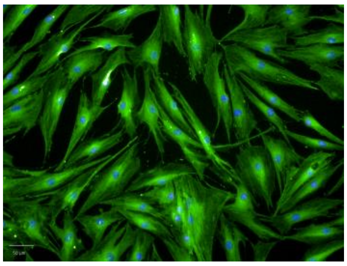

Dex 50 nM:

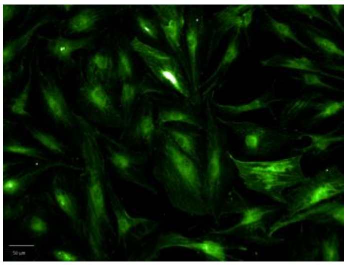

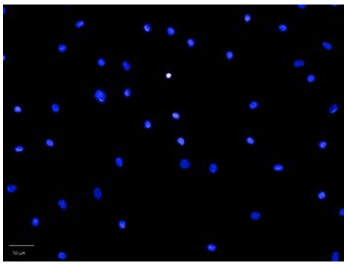

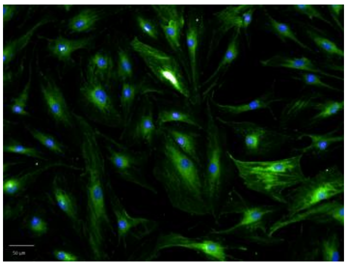

Gent 25 μM:

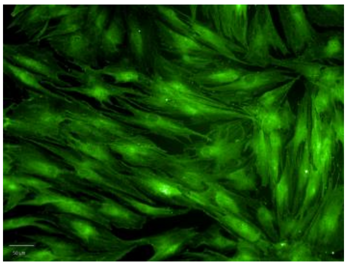

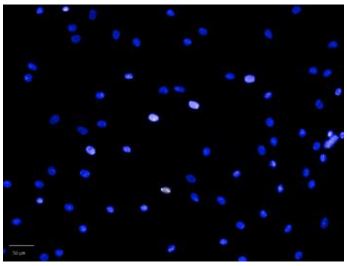

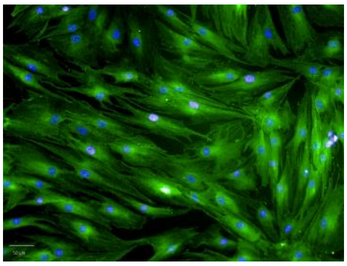

Gent 50 μM:

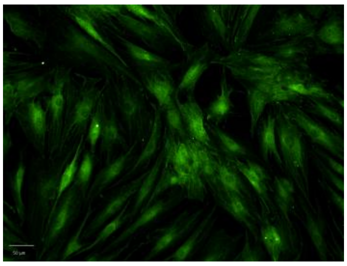

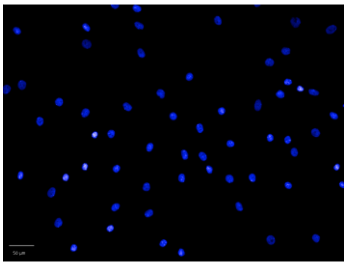

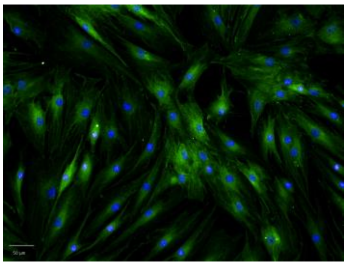

Gent 100 μM:

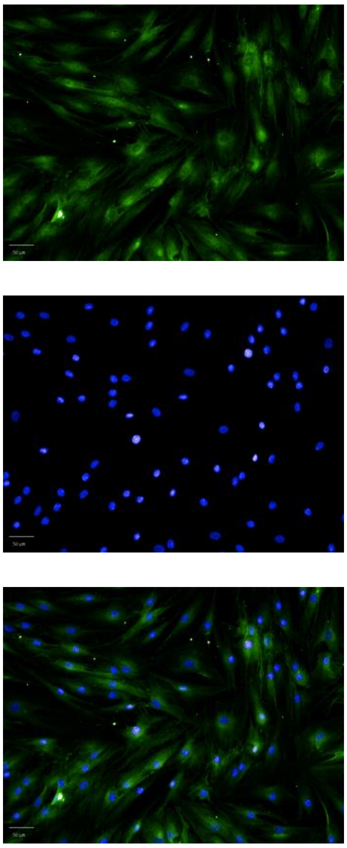

Figure 10C: The original western blots of Figure 10C

NLRP3: Control, TNF-α, Dex 50 nM, Gent 25 μM, Gent 50 μM, Gent 100 μM

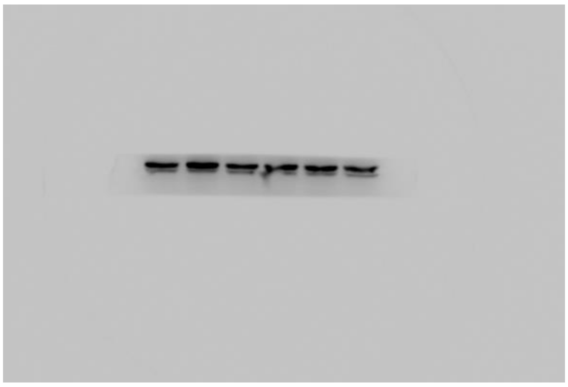

GAPDH: Control, TNF-α, Dex 50 nM, Gent 25 μM, Gent 50 μM, Gent 100 μM

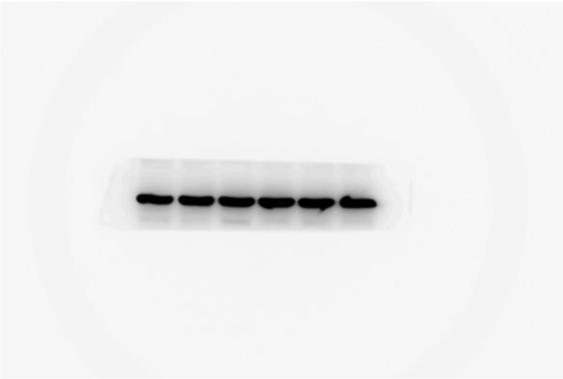

ASC: Control, TNF-α, Dex 50 nM, Gent 25 μM, Gent 50 μM, Gent 100 μM

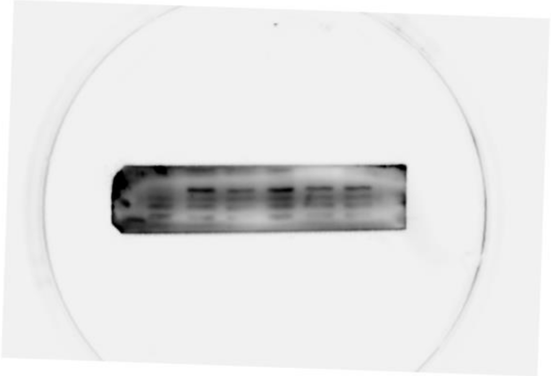

GAPDH: Control, TNF-α, Dex 50 nM, Gent 25 μM, Gent 50 μM, Gent 100 μM

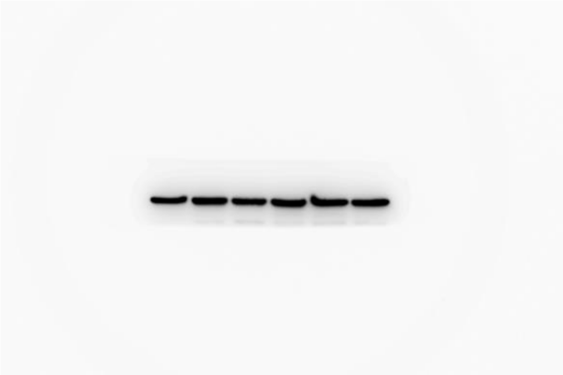

Caspase-1: Control, TNF-α, Dex 50 nM, Gent 25 μM, Gent 50 μM, Gent 100 μM

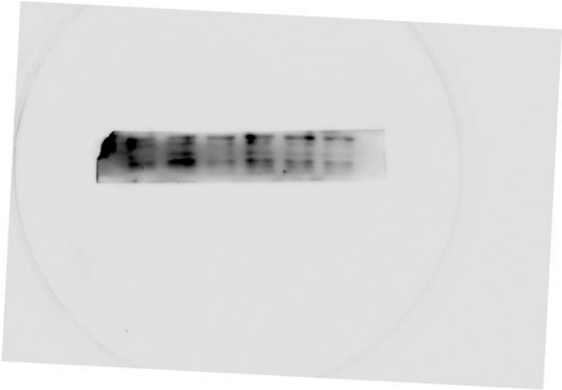

GAPDH: Control, TNF-α, Dex 50 nM, Gent 25 μM, Gent 50 μM, Gent 100 μM

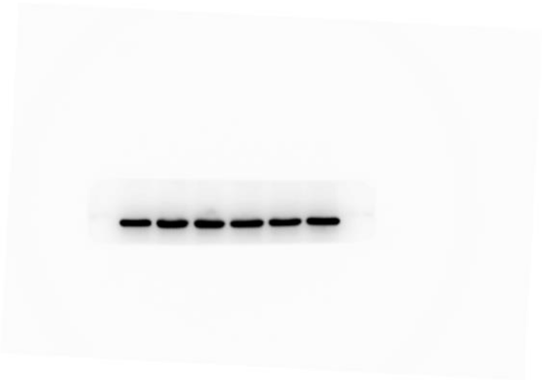

Supplement: Supplementary file 3 [file DataSheet_3.pdf]
